# The impact of coronary artery disease risk loci on ischemic heart failure severity and prognosis: association analysis in the COntrolled ROsuvastatin multiNAtional trial in heart failure (CORONA)

**DOI:** 10.1186/s12881-014-0140-3

**Published:** 2014-12-21

**Authors:** Vincent G Haver, Niek Verweij, John Kjekshus, Jayne C Fox, Hans Wedel, John Wikstrand, Wiek H van Gilst, Rudolf A de Boer, Dirk J van Veldhuisen, Pim van der Harst

**Affiliations:** University of Groningen, University Medical Center Groningen, Department of Cardiology, Hanzeplein 1, 9713GZ Groningen, The Netherlands; Department of Cardiology, University of Oslo and Oslo University Hospital, Oslo, Norway; AstraZeneca Pharmaceuticals, Alderley Park, Macclesfield, Cheshire SK10 4TG UK; Nordic School of Public Health, Göteborg, Sweden; Wallenberg Laboratory for Cardiovascular Research, Sahlgrenska Academy, Gothenburg University, Gothenburg, Sweden; University of Groningen, University Medical Center Groningen, Department of Genetics, Hanzeplein 1, 9713GZ Groningen, The Netherlands; Durrer Center for Cardiogenetic Research, ICIN-Netherlands Heart Institute, 3511GC Utrecht, The Netherlands

**Keywords:** Coronary artery disease, Heart failure, Genetics, Healthy ageing, SNP

## Abstract

**Background:**

Recent genome-wide association studies have identified multiple loci that are associated with an increased risk of developing coronary artery disease (CAD). The impact of these loci on the disease severity and prognosis of ischemic heart failure due to CAD is currently unknown.

**Methods:**

We undertook association analysis of 7 single nucleotide polymorphism (rs599839, rs17465637, rs2972147, rs6922269, rs1333049, rs501120, and rs17228212) at 7 well established CAD risk loci (1p13.3, 1q41, 2q36.3, 6q25.1, 9p21.3, 10q11.21, and 15q22.33, respectively) in 3,320 subjects diagnosed with systolic heart failure of ischemic aetiology and participating in the COntrolled ROsuvastatin multiNAtional Trial in Heart Failure (CORONA) trial. The primary outcome was the composite of time to first event of cardiovascular death, non-fatal myocardial infarction and non-fatal stroke, secondary outcomes included mortality and hospitalization due to worsening heart failure.

**Results:**

None of the 7 loci were significantly associated with the primary composite endpoint of the CORONA trial (death from cardiovascular cases, nonfatal myocardial infarction, and nonfatal stroke). However, the 1p13.3 locus (rs599839) showed evidence for association with all-cause mortality (after adjustment for covariates; HR 0.74, 95%CI [0.61 to 0.90]; *P* = 0.0025) and we confirmed the 1p13.3 locus (rs599839) to be associated with lipid parameters (total cholesterol (*P* = 1.1x10^−4^), low-density lipoprotein levels (*P* = 3.5 × 10^−7^) and apolipoprotein B (*P* = 2.2 × 10^−10^)).

**Conclusion:**

Genetic variants strongly associated with CAD risk are not associated with the severity and outcome of ischemic heart failure. The observed association of the 1p13.3 locus with all-cause mortality requires confirmation in further studies.

**Electronic supplementary material:**

The online version of this article (doi:10.1186/s12881-014-0140-3) contains supplementary material, which is available to authorized users.

## Background

Heart Failure (HF) is a highly prevalent condition affecting more than 15 million patients in Europe alone and has a poor prognosis [1]. In several familial forms of HF, disease-causing mutations have been identified including several mutations in genes coding for structural components of the sarcomere [2,3]. For example, mutations in the genes encoding cardiac β-myosin heavy chain (*MYH7*) or cardiac myosin-binding protein C (*MYBPC3*) are known to cause hypertrophic and dilated cardiomyopathies [4]. However, these Mendelian diseases only account for a small minority of all HF cases and only explain a minor proportion of the population attributable risk for HF. The vast majority of HF is a consequence of more complex genetic and environmental factors and the interactions among them. Coronary artery disease (CAD) is considered the major cause of HF. For the development of CAD, a number of genetic risk loci with common variants have recently been identified [5-7]. The earliest findings derived from genome wide association studies were reported by the Wellcome Trust Case Control Consortium (WTCCC) and the German MI Family GWA studies, as well as the Coronary Artery Disease consortium, which together have identified 7 common variants that were robustly associated with CAD [5,7].

Whether these variants, with strong prior evidence to be associated with increased CAD risk, are also relevant for ischemic HF progression as reflected by HF severity and prognosis remains to be determined. In the present study, we have evaluated these 7 CAD risk loci in ischemic HF patients participating in the COntrolled ROsuvastatin multiNAtional study in heart failure (CORONA) and tested the hypothesis that the SNPs associated with CAD are also associated with ischemic HF disease severity and outcome.

## Methods

The current study is a genetic sub-study of the CORONA trial. The CORONA trial aimed to determine the effect of rosuvastatin treatment on clinical outcome in patients with systolic heart failure from ischemic aetiology. The CORONA study group is represented in the supplement. The CORONA trial has been published in detail, the main result was that rosuvastatin did not reduce the primary composite outcome of death from cardiovascular causes, nonfatal myocardial infarction, or nonfatal stroke [8,9].

### Study population

In- and exclusion criteria of the CORONA trial have been reported in detail previously [8,9]. In brief, patients were 60 years of age or older, HF of ischemic aetiology, and a left ventricular ejection fraction (LVEF) of ≤ 40% with NYHA class III/IV symptoms or LVEF ≤ 35% with NYHA class II symptoms. Patients had to be clinically stable and on optimal treatment for at least 2 weeks before inclusion. Exclusion criteria included the following; recent myocardial infarction (MI) (<6 months); unstable angina or stroke within the past 3 months; percutaneous coronary intervention (PCI) or coronary-artery bypass grafting (CABG); the implantation of a cardioverter defibrillator (ICD) or biventricular pacemaker within the past 3 months; heart transplantation; clinically significant/uncorrected primary valvular heart disease; hypertrophic cardiomyopathy; or systemic disease (e.g., amyloidosis). The CORONA trial included 5,011 patients [9], and DNA was obtained from 3,340 of them. We excluded 20 non-Caucasian subjects (8 black, 7 Asian, 5 ‘other’), leaving 3,320 subjects for the current genetic sub-study of CORONA. The ethical committee at each of the participating hospitals approved this trial (see Additional file 1), and patients provided written informed consent.

### Definition of phenotypes and outcome

The severity of HF was assessed by LVEF and serum N-terminal pro B-type Natriuretic Peptide (NT-proBNP) levels. As one of the loci has also been identified in a GWAS for lipid traits, we specifically studied the association of this locus with available lipid traits. The primary outcome of the CORONA trial was the composite of death from cardiovascular cases, nonfatal myocardial infarction, and nonfatal stroke, analysed according to the time to the first event. Secondary outcomes were death from any cause, any coronary event (defined as sudden death, fatal or nonfatal myocardial infarction, the performance of PCI or CABG, ventricular defibrillation by an ICD, resuscitation after cardiac arrest, or hospitalization for unstable angina), death from cardiovascular causes, sudden death, death from worsening heart failure, combination of mortality or worsening heart failure and athero-thrombotic endpoint.

### SNP selection and genotyping

We studied 7 loci (1p13.3, 1q41, 2q36.3, 6q25.1, 9p21.3, 10q11.21, 15q22.33) which have been linked to CAD risk by previous GWAS [5,7]. We genotyped the 7 lead SNPs within these loci; rs599839, rs17465637, rs2972147, rs6922269, rs1333049, rs501120, and rs17228212 (Additional file 2: Table S1). Genotyping was carried out with TaqMan (Applied Biosystems) using standard protocols and was performed at the laboratory of AstraZeneca Pharmaceuticals (JCF), Alderley Park, UK without knowledge of clinical data.

### Statistical analysis

Normality of the data was determined by visual inspection. HsCRP and NT-proBNP were non-normally distributed and therefore log-transformed. Genotypes were coded additively as 0, 1 or 2 in terms of the number of minor alleles. The baseline variables were analysed in linear model with only genotype as predictor and if significant, the following covariates were added to the adjusted model: age, sex, ejection fraction, NYHA class, systolic blood pressure, heart rate, body mass index, history of myocardial infarction, angina pectoris, diabetes mellitus, hypertension, stroke, intermittent claudication, aortic aneurysm, percutaneous coronary intervention, coronary artery bypass graft surgery, atrial fibrillation, implanted pacemaker, implanted cardiac defibrillator, smoking status, serum creatinine, alanine aminotransferase, creatine kinase, thyroid-stimulating hormone, triglycerides, hsCRP and NT-proBNP, as explained in detail by Wedel *et al*. [10]. HF outcome determinants were analysed using Cox regression of outcome versus number of minor alleles. Analyses were conducted unadjusted and after adjusted for the above mentioned co-variates. We considered a *P*-value <0.0071 for the primary endpoint statistically significant (Bonferroni adjustment of <0.05 for 7 independent loci) and as suggestive for all secondary endpoint analyses.

## Results

The baseline characteristics of the study population are presented in Table 1. The study population consisted of 3,320 HF patients with mean LVEF of 31% and a median NT-proBNP level of 151 pmol/L. There was a high prevalence of hypertension, diabetes mellitus, and chronic kidney disease in our population. Subjects were well treated for HF, with 87% using diuretics, 77% using beta-blockers, 92% using ACE inhibitors or AT1-receptor antagonist, and 91% were treated with an antiplatelet agent or anticoagulant. The mean follow-up time was 2.73 years, accumulating 9,062 patient-years of follow-up. At baseline, 1,986 patients (60%) had a history of MI, and 823 (25%) a history of CABG or PCI. Genotyping was successful in excess of 98% for all SNPs but one and the distributions of genotypes were consistent with the Hardy-Weinberg equilibrium as calculated using the chi-square test for deviation. Minor allele frequencies were similar in the current analyses compared to the discovery GWAS [5,7]. Details on genotyping can be found in Additional file 2: Table S1.

**Table 1:** **Baseline characteristics of subjects in genetic sub-study of CORONA**

**Characteristics of patients**	***n*** **= 3,320**
Age (years)	72.3 ± 6.9
Males *n* (%)	2530 (76.2)
Left ventricular ejection fraction (% ± SD)	31 ± 6.3
NYHA class *n* (%)	
II	1251 (37.7)
III	2035 (61.3)
IV	34 (1.0)
History of *n* (%)	
Angina Pectoris	2463 (74.2)
Aortic Aneurysm	84 (2.5)
Aortic Aneurysm Surgery Performed	47 (1.4)
Atrial Fibrillation/Flutter	1318 (39.7)
Diabetes Mellitus	933 (28.1)
Hypertension	2173 (65.5)
Implantable cardioverter-defibrillator	79 (2.4)
Implanted pacemaker	349 (10.5)
Intermitted claudication	392 (11.8)
Myocardial infarction	1986 (59.8)
Coronary Artery Bypass Surgery	537 (16.2)
Percutaneous Coronary Intervention	358 (10.8)
CABG or PCI	823 (24.8)
Stroke	386 (11.6)
Smoking status *n* (%)	
Non Smoker	1521 (45.8)
(Ex-)smoker	1797 (54.1)
Heart Failure Medication at baseline *n* (%)	
Loop diuretic	2421 (72.9)
Thiazide diuretic	776 (23.4)
Loop or Thiazide	2879 (86.7)
Beta-Blocker	2542 (76.6)
ACE inhibitor	2696 (81.2)
AT1-receptor blocker	428 (12.9)
ACE inhibitor or AT1-receptor blocker	3063 (92.3)
Aldosterone antagonist	1284 (38.7)
Digitalis	1072 (32.3)
Anti-platelet or Anti-coagulant	3020 (91.0)
Blood pressure (mmHg)	
Systolic	130.5 ± 16.1
Diastolic	77.0 ± 8.6
Heart rate (beats/min)	71.2 ± 10.9
BMI (kg/m^2^)	27.5 ± 4.4
Serum creatinine (umol/L)	112.8 ± 26.5
eGFR (ml/min/1.73 m/m^2^BSA)	58.5 ± 14.0
hs-CRP (mg/L)	3.3 (0.02-230)
NT-proBNP (pmol/L)	151 (1–3868)
Lipids	
Total cholesterol (mmol/L)	5.41 ± 1.07
LDL-cholesterol (mmol/L)	3.60 ± 0.94
HDL-cholesterol (mmol/L)	1.19 (0.47-3.55)
Apo-A1 (g/L)	1.51 ± 0.27
Apo-B (g/L)	1.28 ± 0.30
Apo-B/Apo-A (mean)	0.87 ± 0.24
Triglycerides (mmol/L)	1.68 (0.41-14.43)

*Abbreviations:* *NYHA* New York Heart Association, *CABG* Coronary Artery Bypass Graft, *PCI* Percutaneous Coronary Intervention, *ACE* Acetylcholinesterase, *AT1* Angiotensin-1, *BMI* body mass index, *eGFR* estimated Glomerular Filtration Rate, *hs-CRP* high sensitive C-reactive protein, *NT-proBNP* N-terminal pro B-type natriuretic peptide, *LDL* low-density lipoprotein, *HDL* high-density lipoprotein, *Apo* apolipoprotein. Variables are expressed as mean (SD) when normally distributed and as median (min-max) when non-normally distributed.

### CAD loci and HF disease severity; LVEF and NT-proBNP

LVEF and NT-proBNP were taken as indicators of HF disease severity and their association with the 7 genetic loci was determined. Although some of the unadjusted association *P*-values were smaller than 0.05 (Table 2, Additional file 3: Table S2, and Additional file 4: Table S3), we considered none of these loci associated with LVEF or NT-proBNP considering the multiple testing burden of these secondary endpoints.

**Table 2:** **CAD loci (** ***P*** **< 0.05) and heart failure disease markers in genetic sub-study of CORONA**

	**Locus**	**SNP**	**Model**	***n***	**Beta estimate**	**95% CI**	***P*** **value**	**Excluded from adjusted analysis**
LVEF	9p21.3	rs1333049	Unadj.	3,300	0.0044	0.0015 - 0.0074	3.6 × 10^−3^	LVEF
			Adj.^a^	2,212	0.0038	0.0005 - 0.0071	2.3 × 10^−2^	
	10q11.21	rs501120	Unadj.	3,300	0.0054	0.0012 - 0.0097	1.2 × 10^−2^	
			Adj.^a^	2,216	0.0022	−0.0025 - 0.0068	0.36	
	15q22.33	rs17228212	Unadj.	3,303	−0.005	−0.0084 - -0.0016	4.0 × 10^−3^	
			Adj.^a^	2,218	−0.0031	−0.0069 - 0.0008	0.12	
log NT-proBNP (pmol/L)	10q11.21	rs501120	Unadj.	2,412	−0.11	−0.20 - -0.01	2.7 × 10^−2^	log NT-proBNP (pmol/L)
			Adj.^a^	2,216	−0.064	−0.14 - 0.02	0.12	
BMI (kg/m2)	1q41	rs17465637	Unadj.	3,298	0.35	0.11 - 0.60	4.8 × 10^−3^	BMI (kg/m2)
			Adj.^a^	2,220	0.3	0.03 - 0.58	2.9 × 10^−2^	
Serum creatinine (umol/L)	10q11.21	rs501120	Unadj.	3,300	−2.01	−3.80 - -0.21	2.8 × 10^−2^	Serum creatinine (umol/L)
			Adj.^a^	2,216	−0.43	−2.25 - 1.39	0.65	

*Abbreviations:* *SNP* single nucleotide polymorphism, *LVEF* left ventricular ejection fraction, *BMI* body mass index, *NT-proBNP* N-terminal pro B-type natriuretic peptide. ^a^Adjusted analyses were adjusted for age, sex, ejection fraction, NYHA class, systolic blood pressure, heart rate, body mass index, history of myocardial infarction, angina pectoris, diabetes mellitus, hypertension, stroke, intermittent claudication, aortic aneurysm, percutaneous coronary intervention, coronary artery bypass graft surgery, atrial fibrillation, implanted pacemaker, implanted cardiac defibrillator, smoking status, serum creatinine, alanine aminotransferase, creatine kinase, thyroid-stimulating hormone, triglycerides, hsCRP and NT-proBNP [10]. As some covariates were also baseline variables or strongly associated to a baseline variable, covariates were excluded from analyses (see Additional file 3: Table S2). Results of all regression analyses for all SNPs are in Additional file 4: Table S3.

### Prognostic value of CAD loci for cardiovascular events and disease progression in HF

Next, we tested the association between the CAD-associated loci with HF disease outcome. None of the 7 loci predicted the occurrence of the primary endpoint (composite endpoint of cardiovascular mortality, non-fatal myocardial infarction or non-fatal stroke, analysed as time to first event) or death caused by cardiovascular events. When the individual components of the primary endpoint were considered, we observed that the 1p13.3 (rs599839) locus, showed a borderline association with all-cause mortality (HR 0.86, 95% CI [0.74-1.00], *P* = 0.499) which became somewhat stronger after adjustment for covariates (HR 0.74, [0.61-0.90], *P* = 0.0025). Using ordered Jonckheere-Terpstra test, the 1p13.3 locus also showed association with the total number of hospitalizations due to cardiovascular causes (*P* = 0.0093) and the 10q11.21 locus showed an association with the number of hospitalizations due to worsening HF. All associations with *P* < 0.05 are presented in Table 3 (all associations are presented in Additional file 5: Table S4 and Additional file 6: Table S5.

**Table 3:** **CAD loci (** ***P*** **< 0.05) and prognosis of ischemic heart failure in genetic sub-study of CORONA**

	**Locus**	**SNP**	**Model**	***n*** **(total)**	***n*** **(events)**	**Hazard ratio**	**95% CI**	***P*** **value**
All-cause Mortality	1p13.3	rs599839	Unadj.	3,300	527	0.86*	0.74-1.00	4.99 × 10^−2^
			Adj.^a^	2,218	341	0.74*	0.61-0.90	2.5 × 10^−3^
Mortality or WHF hospitalization	10q11.21	rs501120	Unadj.	3,300	1046	0.85*	0.75-0.97	1.2 × 10^−2^
			Adj.^a^	2,216	670	0.82*	0.70-0.96	1.5 × 10^−2^
Number of hospitalizations due to cardiovascular cause	1p13.3	rs599839	Ordered Jonkeheere-Terpstra test					9.3 × 10^−3^
Number of hospitalizations due to WHF	10q11.21	rs501120	Ordered Jonkeheere-Terpstra test					3.2x10^−2^

*Abbreviations: SNP* single nucleotide polymorphism, *WHF* worsening heart failure. ^a^Adjusted analyses were adjusted for age, sex, ejection fraction, NYHA class, systolic blood pressure, heart rate, body mass index, history of myocardial infarction, angina pectoris, diabetes mellitus, hypertension, stroke, intermittent claudication, aortic aneurysm, percutaneous coronary intervention, coronary artery bypass graft surgery, atrial fibrillation, implanted pacemaker, implanted cardiac defibrillator, smoking status, serum creatinine, alanine aminotransferase, creatine kinase, thyroid-stimulating hormone, triglycerides, hsCRP and NT-proBNP [10]. *directions were concordant with previous observations. [7] Regression data of all SNPs are presented in Additional file 5: Table S4.

### Associations of CAD loci with lipid profile in HF

The 7 loci were evaluated for association with the available serum lipid profile parameters. After adjustments, the 1p13.3 locus (rs599839) was associated with total cholesterol (*P* = 1.1 × 10^−4^), low-density-lipoprotein (LDL) cholesterol (*P* = 3.5 × 10^−7^), serum Apolipoprotein-B (Apo-B) levels (*P* = 5.1 × 10^−8^) and the Apo-B/Apo-A1 ratio (*P* = 8.0 × 10^−9^). Associations with lipid parameters with *P*-values smaller than 0.05 are presented in Table 4 (all associations are presented in Additional file 7: Table S6.

**Table 4:** **CAD loci (** ***P*** **< 0.05) and lipid characteristics in genetic sub-study of CORONA**

	**Locus**	**SNP**	**Model**	***n***	**Beta estimate**	**95% CI**	***P*** **value**
Total cholestorol (mmol/L)	1p13.3	rs599839	Unadj.	3,285	−0.14	−0.20 - -0.08	1.2 × 10^−5^
			Adj.^a^	2,219	−0.14	−0.21 - -0.07	1.1 × 10^−4^
	10q11.21	rs501120	Unadj.	3,285	−0.07	−0.15 - -0.001	4.7 × 10^−2^
			Adj.^a^	2,217	−0.05	−0.13 - 0.03	0.24
LDL cholesterol (mmol/L)	1p13.3	rs599839	Unadj.	3,285	−0.16	−0.22 - -0.11	1.8 × 10^−9^
			Adj.^a^	2,219	−0.17	−0.23 - -0.1	3.5 × 10^−7^
	10q11.21	rs501120	Unadj.	3,285	−0.07	−0.13 - -0.004	3.6 × 10^−2^
			Adj.^a^	2,217	−0.05	−0.12 - 0.02	0.19
Triglycerides (mmol/L)	1p13.3	rs599839	Unadj.	3,285	0.08	0.007 - 0.15	3.2 × 10^−2^
			Adj.^a^	2,219	0.07	−0.005 - 0.15	6.6 × 10^−2^
	9p21.3	rs1333049	Unadj.	3,285	−0.08	−0.14 - -0.02	7.0 × 10^−3^
			Adj.^a^	2,213	−0.06	−0.13 - 0.001	5.4 × 10^−2^
Apo-B (g/L)	1p13.3	rs599839	Unadj.	3,264	−0.06	−0.07 - -0.04	2.2 × 10^−10^
			Adj.^a^	2,218	−0.06	−0.08 - -0.04	5.1 × 10^−8^
	10q11.21	rs501120	Unadj.	3,267	−0.02	−0.04 - -0.002	2.8 × 10^−2^
			Adj.^a^	2,216	−0.02	−0.05 - 0.002	7.5 × 10^−2^
Apo-B/Apo-A1 ratio	1p13.3	rs599839	Unadj.	3,264	−0.05	−0.06 - -0.03	3.3 × 10^−11^
			Adj.^a^	2,218	−0.05	−0.07 - -0.03	8.0 × 10^−9^

*Abbreviations: SNP* single nucleotide polymorphism, *LDL* low-density-lipoprotein, *HDL* high-density-lipoprotein, *Apo-B* apolipoprotein-B, *Apo-A1* apolipoprotein-A1. ^a^Adjusted analyses were adjusted for age, sex, ejection fraction, NYHA class, systolic blood pressure, heart rate, body mass index, history of myocardial infarction, angina pectoris, diabetes mellitus, hypertension, stroke, intermittent claudication, aortic aneurysm, percutaneous coronary intervention, coronary artery bypass graft surgery, atrial fibrillation, implanted pacemaker, implanted cardiac defibrillator, smoking status, serum creatinine, alanine aminotransferase, creatine kinase, thyroid-stimulating hormone, triglycerides, hsCRP and NT-proBNP [10]. As some covariates were also baseline variables or strongly associated to a baseline variable, covariates were excluded from analysis (see Additional file 3: Table S2). Data for all SNPs are presented in Additional file 7: Table S6.

## Discussion

HF is a common condition in which cardiac function is affected, leading to a variety of symptoms like dyspnoea, fatigue, and fluid retention. The most frequent cause of HF is CAD. In the past few years, several genetic variants have been associated with CAD resulting in new insight in the pathophysiology of CAD [5]. We tested the hypothesis that the presence of variants associated with prevalent CAD is also linked to increased severity of HF and worse prognosis. Our findings lend little support to this hypothesis. This was a candidate variant analyses and some of the observed associations are suggestive and require further replication.

The loci we evaluated included the 9p21 locus, which is considered the most robust common genetic risk factor for CAD with the rs1333049 variant showing the strongest signal for association with CAD in WTCCC and German studies [5]. This locus harbours a large non-coding transcript of unknown function, designated the name CDKN2B antisense RNA (CDKN2BAS or ANRIL). This locus is related to atherosclerotic disease burden in different vascular beds [11] and deletion of ANRIL in human aortic smooth muscle cells leads to a increase in proliferative capacity in culture [12]. Furthermore, the rate of proliferation of vascular smooth muscle cells is attenuated by the 9p21 genotype and the CAD risk allele (C allele) increases vascular smooth muscle cell proliferation, thereby likely playing an important role in the development of atherosclerosis [13]. Despite these findings, the presence of this genetic variant, and presumably increased atherosclerotic burden, did not translate into increased HF severity or worse outcome in patients with ischemic HF in the present study. A possible explanation is that the effect of this locus acting through increased atherosclerotic disease burden is confounded by events defined by the severity of heart failure. In addition, the vulnerability to develop HF given a similar atherosclerotic disease burden might also differ among patients and could be a consequence of complex gene-environment interactions. The presence or absence of concomitant conditions and diseases including diabetes, renal dysfunction, or pulmonary disease may also modify the phenotype. Furthermore, patients in our study were well treated with beta-blockers, angiotensin-converting-enzyme inhibitors, and aldosterone antagonists, which might further have decreased the differences between patients due to genetic risk factors. Also related to optimisation of pharmacotherapy, Wedel *et al*. calculated specifically for the CORONA trial, that the proportionate contribution of myocardial infarction to total mortality, related to the atherosclerotic disease burden, was relatively small. The Wald statistic of myocardial infarction for total mortality was 3.9, while for example log NT-proBNP showed a Wald statistic for total mortality of 167 [10]. This suggests that the pathological role of genetic risk variants acting through CAD might also be relatively small, and could therefore have remained undetected in our study. Nevertheless, the current sample size of systematically collected ischemic HF patients with DNA available to perform genotyping studies is the largest available in the world to date and it will be difficult, if not impossible, to extend the sample size within reasonable timeframes. The results of this study concurs with the lack of effect on CAD with rosuvastatin [10].

Considering prior knowledge, we also studied the association of the genotyped genetic variants with lipid parameters [14-16]. We were able to confirm previous reported associations between the 1p13.3 locus (rs599839) and lipids. We also provided novel data on this locus and its association with apolipoproteins. The 1p13.3 locus was most strongly associated with Apo-B levels (stronger than the known LDL association), suggesting Apo-B is a candidate effector molecule. The genomic region at 1p13.3 encodes 4 genes: proline/serine-rich coiled protein 1 (*PSRC1*), cadherin EGF LAG sevenpass G-type receptor 2 (*CELSR2*), myosin-binding protein H-like (*MYBHL*), and sortilin 1 (*SORT1*). Recent studies have revealed a role for *SORT1* in Apo-B secretion and LDL catabolism: overexpression of *SORT1* resulted in increased serum LDL and loss-of-function mutations were associated with protection against hypercholesterolemia and atherosclerosis [17,18]. In our secondary analyses we observed an association with all-cause mortality and the number of hospitalizations due to cardiovascular causes for the 1p13.3 locus. As these were secondary analyses, which would not survive strict adjustment for multiple testing, the interpretation of these findings should be cautious as they require further confirmation. In addition, the explanation and possible relation to lower cholesterol and apolipoprotein levels cannot be deducted from the current study.

Among the strengths of the present study are the size and quality of the study cohort, which is the largest heart failure cohorts with DNA available to date. Patient characteristics and outcomes have been collected and documented systematically within the framework of a clinical trial. However, there are some limitations which we need to address. At first, we have limited the variants tested to the N.J. Samani paper published in 2007 [5] and the variants reported by the Coronary Artery Disease Consortium in 2009 [7]. Recent GWA studies have identified additional variants involved in CAD development. The recent publication by the CARDIoGRAMplusC4D consortium reported 46 genetic variants associated with CAD risk [19]. We cannot exclude that there might be variants among these 46 that are related to heart failure outcomes and this remains an objective for further study. We performed a post-hoc power calculation to consider the possibility that our study might have lacked power [20]. Assuming an effect size of the risk variant comparable to the CAD discovery GWAS (genotype relative risk Aa = 1.3; genotype relative risk AA = 1.6) [5,7], we calculated the power to detect a significant effect for a variant (prevalence cases = 0.175; number of cases = 581; control:case ratio = 4.7). The power of our analyses ranged from 0.90 to 0.91 depending on the frequency of the risk allele (ranging from 0.25 to 0.55). Future studies with larger sample sizes, containing all CAD loci, are warranted to further evaluate the influence of CAD genetic loci in heart failure.

## Conclusions

Genetic variants associated with CAD and atherosclerotic disease burden are not associated with the severity and prognosis of patients with ischemic HF in the CORONA trial. The observed secondary association of the 1p13.3 locus with all-cause mortality requires confirmation in further studies.

## Additional files

Additional file 1: Supplemental Methods. Independent Ethics Committees/Institutional Review Boards consulted. Additional file 2: Table S1. Genotyping details in the genetic sub-study of CORONA. Additional file 3: Table S2. Covariates in statistical analysis. Additional file 4: Table S3. CAD loci and heart failure disease markers in genetic sub-study of CORONA. Additional file 5: Table S4. CAD loci and prognosis of ischemic heart failure in genetic sub-study of CORONA. Additional file 6: Table S5. CAD loci and hospitalizations of ischemic heart failure in genetic sub-study of CORONA. Additional file 7: Table S6. CAD loci and lipid characteristics in genetic sub-study of CORONA.

## Abbreviations

CAD: Coronary artery disease
CORONA: Controlled Rosuvastatin multinational study in heart failure
HF: Heart failure
HR: Hazard ratio
