# Supplementary material for: The impact of coronary artery disease risk loci on ischemic heart failure severity and prognosis: association analysis in the COntrolled ROsuvastatin multiNAtional trial in heart failure (CORONA)
Source: BMC Med Genet. 2014 Dec 21;15:140. doi: 10.1186/s12881-014-0140-3 (PMC4412120; doi:10.1186/s12881-014-0140-3)
Supplement: Additional file 1: Supplemental Methods. — Independent Ethics Committees/Institutional Review Boards consulted. [file 12881_2014_140_MOESM1_ESM.xlsx]

Supplemental Methods

Independent Ethics Committees/Institutional Review Boards consulted

Centre no.	Name and address of IEC/IRB	Chairman of IEC/IRB	Date of Approval
Belgium<br>101	Faculteit Geneeskunde K.U. Leuven, Herestraat 49<br>BE-3000 Leuven.	P. J. De Schepper	CSP - 15 Aug 2003<br>Amendment no. 1 – 23 Jan 2004<br>Amendment. no. 2 & 3 – 28 Feb 2005
102	AZ Sint Jan AV – Brugge<br>Ruddershove 10<br>BE-8000 Brugge	Guido Willemarck<br><br>J. Van Droogenbroeck	CSP & Amendment no. 1 – 29 Jan 2004<br>Amendment no. 2 & 3 - 3 March 2005
103	ZOL Campus ST. Jan<br>Schoepse Bos 6<br>BE-3600 Genk	E. de Jonge	CSP – 18 Sep 2003<br>Amendment no. 1, 2, 3 – 22 Feb 2005
104	Onze Lieve Vrouwziekenhuis<br>Moorselbaan 164<br>BE–9300 Aalst	A. Leloup	CSP – 23 Oct 2003<br>Amendment no. 1 – 15 April 2004<br>Amendment no. 2 & 3 – 12 April 2005
105	UZA<br>Wilrijkstraat 10<br>BE–2650 Edegem	P. Cras	CSP – 16 Dec 2003<br>Amendment no. 1 – 13 Jan 2004<br>Amendment no. 2 & 3 – 22 Feb 2005
106	Sint Elisabethziekenhuis<br>Rubbensstraat 166 BE–2300 Turnhout	K. S. Ackaert	CSP - 1 Dec 2003<br>Amendment no. 1 – 29 Jan 2004<br>Amendment no. 2 & 3 – 23 March 2005
107	CHR La Tourelle<br>Rue du Parc 29 BE–4800 Verviers	J. L. Corhay<br>G. Cornet	CSP – 3 Nov 2003<br>Amendment no 1 – 16 Nov 2003<br>Amendment 2 & 3 – 22 March 2005
108	Centre Hospitalier HUTOIS<br>Rue 3 Ponts 2<br>BE–4500 Huy	Ph. Mathieu	CSP – 18 Nov 2003,<br>Amendment no. 1 - 16 March 2004<br>Amendment no. 2 – 14 June 2005<br>Amendment no. 3 – 17 Jan 2005

Centre no.	Name and address of IEC/IRB	Chairman of IEC/IRB	Date of Approval
109	Clinique St. Luc<br>Rue St. Luc 8<br>BE–5004 Bouge	Joseph Jaucot CSP	– 25 Nov 2003<br>Amendment no. 1 –<br>21 Dec 2004<br>Amendment no. 2 & 3<br>– 3 May 2005
111	Polyclinique de Fléron<br>Rue Reine Astrid 20<br>BE–4620 Fléron	Henri Pirenne	CSP – 7 Nov 2003<br>Amendment no. 1 –<br>27 Jan 2005<br>Amendment no. 2 & 3<br>– 25 April 2005
112	Kliniek Heilig Hart, Dr. Elshot<br>Cardiology<br>Gasthuisstraat 1<br>BE–2400 Mol	J. W. Verstraten	CSP – 15 April 2004<br>Amendment no. 1 –<br>4 Jan 2005<br>Amendment no. 2 & 3<br>– 23 June 2005
Bulgaria<br>201	Ethics Committee at<br>Multispecialised Hospital for<br>Active Treatment - Pleven<br>20 St. Kiril I Metoddi Str<br>BG–5800 Pleven	Yavor Ivanov	CSP – 8 July 2003<br>Amendment no. 1 –<br>5 Nov 2003<br>Amendment no. 2 & 3<br>– 2 March 2005
202	Ethics Committee at<br>Multispecialised Hospital for<br>Active Treatment – Pleven<br>20 St. Kiril I Metoddi Str<br>BG–5800 Pleven	Yavor Ivanov CSP	– 08 July 2003<br>Amendment no. 1 –<br>20 Jan 2004<br>Amendment no. 2 & 3<br>– 2 March 2005
203, 204	Ethics Committee at<br>Multispecialised Hospital for<br>Active Treatment St. Marina –<br>Varna, 1 Christo Smirnenski Str<br>BG–9000 Varna	Stefan Todorov<br><br><br>Iskren Kotzev	CSP – 16 July 2003<br>Amendment no. 1 –<br>2 Feb 2004<br>Amendment no. 2 & 3<br>– 15 Feb 2005
205	Ethics Committee at<br>Multispecialised Hospital for<br>Active Treatment Ruse<br>2 Nezavisimost Str<br>BG–7002 Russe	P Yankov<br><br><br>Gr. Lefterov	CSP – 21 July 2003<br>Amendment no. 1 –<br>6 June 2004<br>Amendment no. 2 & 3<br>– 21 Feb 2005
206	Ethics Committee at<br>Multispecialised Hospital for<br>Active Treatment Plovdiv<br>234 Bulgaria Blvd<br>BG–4000 Plovdiv	Dr. Tanev<br>Atanas Petrov<br><br>Valentina Spassova	CSP – 9 July 2003<br>Amendment no. 1 –<br>7 Jan 2004<br>Amendment no. 2 & 3<br>– 14 March 2005

Centre no.	Name and address of IEC/IRB	Chairman of IEC/IRB	Date of Approval
207	Ethics Committee at Medical Institute, Central Clinical Hospiltal Sofia<br>79 Gen. Skobelev Blvd<br>BG–1606 Sofia	Dimitar Raev<br>Biserka Penkova<br><br>Mihail Protich	CSP – 4 July 2003<br>Amendment no. 1 – 14 Jan 2004<br>Amendment no. 2 & 3 – 11 Feb 2005
208, 209	Ethics Committee at MHAT Alexandrovska<br>1 Georgi Sofiiski Str BG–1431 Sofia	Jerassi	CSP – 24 July 2003<br>Amendment no. 1 – 8 Jan 2004<br>Amendment no. 2 & 3 – 24 Feb 2005
210	Ethics Committee at Multiprofile Transport Hospital for Active Treatment<br>104 Maria Luiza Blvd<br>BG–1233 Sofia	Haralampi Vasilev	CSP – 11 July 2003<br>Amendment no. 1 – 5 Feb 2004<br>Amendment no. 2 & 3 – 24 March 2005
211	Ethics Committee at National Heart Hospital<br>65 Koniovitza Str BG–1309 Sofia	Margarita Tsonzorova	CSP – 16 July 2003<br>Amendment no. 1 – 4 Feb 2004<br>Amendment no. 2 & 3 – 1 March 2005
212	Ethics Committee at MHAT Alexandrovska<br>1 Georgi Sofiiski Str BG–1431 Sofia	Jerassi	CSP – 24 July 2003<br>Amendment no. 1 – 8 Jan 2004<br>Amendment no. 2 & 3 – 24 Feb 2005
214	Ethics Committee at MHAT Queen Joanna<br>8 Bjalo more Str<br>BG–1504 Sofia	V. Vlahov<br>E. Kolev	CSP – 10 July 2003<br>Amendment no. 1 – 28 Jan 2004<br>Amendment no. 2 & 3 - 28 March 2005
215, 216	Ethiccs Committee at 2nd MHAT<br>120 Hristo Botev Blvd BG–1000 Sofia	Mariana Vasileva	CSP – 30 July 2003<br>Amendment no. 1 – 30 Jan 2004<br>Amendment no. 2 & 3 – 25 Feb 2005
Czech Republic<br>301	EC Vseobecné fakultní nemocnice v Praze<br>Na Bojisti 1<br>CZ–128 08 Prague 2	Hana Vrbová<br>Jiri Kolar	CSP – 03 June 2003<br>Amendment no. 1 – 22 Jan 2004<br>Amendment no. 2 & 3 - 21 April 2005

Centre no.	Name and address of IEC/IRB	Chairman of IEC/IRB	Date of Approval
302	EC FN u sv. Anny in Brno,<br>Pekarská 53<br>CZ–656 91 Brnov	Vladimir Soska	CSP – 5 June 2003<br>Amendment no. 1 – 15 Jan 2004<br>Amendment no. 2 & 3 – 11 March 2005
304	EC Nemocnice Kladno,<br>Vancurova 1548<br>CZ–272 59 Kladno	Petr Prusa<br><br>Tomas Rittstein	CSP – 4 July 2003<br>Amendment no. 1 – 22 Dec 2003<br>Amendment no. 2 & 3 – 25 Feb 2005
306	EC Oblastní Nemocnice<br>Príbram, Podbrdská ulice 269,<br>CZ–261 95 Príbram V - Zdabor	Jan Santora	CSP – 13 June 2003<br>Amendment no. 1 – 12 Dec 2003<br>Amendment no. 2 & 3 – 21 March 2005
308	EC Nemocnice milosrdných<br>sester sv. K. Boromejského,<br>Vlasská 36<br>CZ–110 00 Prague 1	Josef Dosedel	CSP – 25 June 2003<br>Amendment no. 1 – 27 Nov 2003<br>Amendment no. 2 & 3 – 24 May 2005
309	EC Ústrední Vojenské<br>nemocnice u vojenské 1200<br>CZ–169 02 Prague 6	J. Janatka	CSP – 17 June 2003<br>Amendment no. 1 – 15 Dec 2003<br>Amendment no. 2 & 3 – 21 Feb 2005
310	EC FN u sv. Anny in Brno,<br>Pekarská 53<br>CZ–656 91 Brnov	Vladimir Soska<br>Sarka Sedlackova	CSP – 5 June 2003<br>Amendment no. 1 – 14 Jan 2004<br>Amendment no. 2 & 3 – 2 March 2005
311	EC – 3.lekarske fakulty<br>Univerzity Karlovy v Praze<br>Ruská 87<br>CZ–100 00 Prague 10	Jirí Simek	CSP – 23 June 2003<br>Amendment no. 1 – 13 Jan 2004<br>Amendment no. 2 & 3 – 6 June 2005
312	EC of CEPHA s. r. o.<br>Komenského 19<br>CZ-323 00 Plzen	L. Cajzl	CSP – 1 Sep 2003<br>Amendment no. 1 – 15 Dec 2003<br>Amendment no. 2 & 3 – 21 Feb 2005

Centre no.	Name and address of IEC/IRB	Chairman of IEC/IRB	Date of Approval
314	EC Nemocnice Jihlava,<br>Vrchlického 59<br>CZ–586 33 Jihlava	Josef Ehl<br><br><br>Roman Peschout	CSP – 3 Sep 2003<br>Amendment no. 1 – 12 Dec 2003<br>Amendment no. 2 & 3 – 17 March 2005
315	EC Nemocnice Slaný,<br>Politických veznu 576<br>CZ–274 01 Slaný	Bohumil Vasek<br><br><br>Gabriel Marcínek	CSP – 3 June 2003<br>Amendment no. 1 – 16 Jan 2004<br>Amendment no. 2 & 3 – 17 March 2005
316	EC FN Plzen<br>Tr. Dr. E. Benese 13<br>CZ–305 99 Plzen	J. Fínek	CSP – 26 June 2003<br>Amendment no. 1 – 11 Dec 2003<br>Amendment no. 2 & 3 – 24 Feb 2005
317	EC Nemocnice<br>Slovanského bratrství 710<br>CZ–393 01 Pelhrimov	Ondrej Skoda	CSP – 18 June 2003<br>Amendment no. 1 – 17 Dec 2003<br>Amendment no. 2 & 3 – 17 Feb 2005
318	EC Vseobecné fakultní nemocnice v Praze<br>Na Bojisti 1<br>CZ–128 08 Prague 2	R. Pánková	CSP – 3 June 2003<br>Amendment no. 1 – 22 Jan 2004<br>Amendment no. 2 & 3 – 21 April 2005
320	EC Vseobecné fakultní nemocnice v Praze<br>Na Bojisti 1<br>CZ–128 08 Prague 2	Hana Vrbová<br><br>R. Pánková	CSP – 3 June 2003<br>Amendment no. 1 – 22 Jan 2004<br>Amendment no. 2 & 3 – 21 April 2005
322	Regionální EC Okresní Nemocnice Tábor<br>Kpt. Jarose 2000<br>CZ–393 03 Tábor	Kamil Klenha	CSP – 26 June 2003<br>Amendment no. 1 – 23 March 2004<br>Amendment no. 2 & 3 – 10 Mar 2005
323	EC FN Plzen<br>Tr. Dr. E. Benese 13<br>CZ–305 99 Plzen	J. Fínek	CSP – 26 June 2003<br>Amendment no. 1 – 20 May 2004<br>Amendment no. 2 & 3 – 24 Feb 2005

Centre no.	Name and address of IEC/IRB	Chairman of IEC/IRB	Date of Approval
324	EC Nemocnice na Homolce,<br>Roentgenova 2<br>CZ–151 19 Praha 5	Rudolf Ramaisl	CSP – 28 May 2003<br>Amendment no. 1 –<br>15 Jan 2004<br>Amendment no. 2 & 3<br>– 17 Feb 2005
325	EC FN v Motole<br>V Úvalu 84<br>CZ–150 06 Praha 5	Vratislav Smelhaus	CSP – 25 June 2003<br>Amendment no. 1 –<br>17 Dec 2003<br>Amendment no. 2 & 3<br>– 9 Feb 2005
326	EC FN Královské Vinohrady,<br>Srobárova 50<br>CZ–100 34 Praha 10	Jan Pachl	CSP – 3 June 2003<br>Amendment no. 1 –<br>11 Febr 2004<br>Amendment no. 2 & 3<br>– 9 Feb 2005
328	EC Mestská Nemocnice<br>Ostrava, Nemocnicní 20<br>CZ–728 80 Ostrava	Jan Nieslanik	CSP – 19 Aug 2003<br>Amendment no. 1 –<br>16 Dec 2003<br>Amendment no. 2 & 3<br>– 1 Mar 2005
329	EC Nemocnice Liberec<br>Husova 10<br>CZ–460 63 Liberec	Marie Rissová	CSP – 4 June 2003<br>Amendment no. 1 –<br>17 Dec 2003<br>Amendment no. 2 & 3<br>– 23 Feb 2005
330	EC Nemocnice Rudolf and<br>Stefanie Benesov<br>Máchova 400<br>CZ–256 01 Benesov	Jirí Bráza	CSP – 4 June 2003<br>Amendment no. 1 –<br>18 Dec 2003<br>Amendment no. 2 & 3<br>– 23 Mar 2005
Denmark<br>All centres	Den Videnskabsetiske Komité<br>for Köpenhavns og<br>Frederiksberg Kommuner,<br>Köpenhavns<br>Sundhedsdirektorat, Postboks<br>620, Sjaellandsgade 40<br>DK–2200 Köpenhavn N	Rikard Vrogaard	CSP – 12 Aug 2003<br>Amendment no. 1 –<br>26 Feb 2004<br>Amendment no. 2 & 3<br>– 23 Mar 2005
Finland<br>All centres	The Ethics committee of HUS<br>internatioanl diseases,<br>Sisätaudit, PL 340<br>FIN–00029 Hus	Vesa Manninen	CSP – 23 June 2003<br>Amendment no. 1 –<br>16 Dec 2003<br>Amendment no. 2 & 3<br>– 8 June 2005

Centre no.	Name and address of IEC/IRB	Chairman of IEC/IRB	Date of Approval
France<br>All centres	CCPPRB de La Pitié Salpétrière<br>47 Boulevard de l'hôpital<br>FR-75013 Paris	Thierry Hergueta	CSP – 2 July 2003<br>Amendment no. 1 –<br>17 dec 2003<br>Amendment no. 2 & 3<br>– 2 Feb 2005
Germany<br>701	Ärztekammer des Saarlandes<br>Faktoreistr. 4<br>DE–66111 Saarbrücken	Schieffer	CSP – 15 July 2003<br>Amendment no. 1 –<br>7 Jan 2004<br>Amendment no. 2 & 3<br>– 14 April 2005
702	Saechisische Landesaerztekammer, Ethik-Komission, Schuetzenhoehe 16, DE–01099 Dresden	Haupt	CSP – 25 Aug 2003<br>Amendment no. 1 -<br>4 Febr 2004<br>Amendment no. 2 & 3<br>– 3 May 2005
703	Charité, Ethik-Kommission<br>Ethikausschuss am Campus Virchow-Klinikum,<br>Schumannstr. 20/21 DE–10098 Berlin	E. Garbe	CSP – 18 Febr 2004<br>Amendment no. 2 & 3<br>– 20 July 2005
	Charité-Universitätsmedizin<br>Augustenburger Platz 1<br>DE-13353 Berlin	H. Eichstädt	Amendment no. 1 -<br>17 March 2004
705	Ärztekammer Berlin<br>Friedrichstr. 16<br>DE–10969 Berlin	C. Jasper	CSP – 25 Aug 2003<br>Amendment no. 2 & 3<br>– 29 april 2005
	Charité-Universitätsmedizin<br>Augustenburger Platz 1<br>DE-13353 Berlin	H. Eichstädt	Amendment no. 1 -<br>17 March 2004
706	Landesärztekammer Thueringen, Ethik-Kommission<br>Im Semmicht 33<br>DE–07751 Jena	R. Giertler	CSP – 1 Oct 2003<br>Amendment no. 1 -<br>4 Febr 2004<br>Amendment no. 2 & 3<br>– 25 May 2005
707	Saechisische Landesaerztekammer, Ethik-Kommission<br>Schuetzenhoehe 16 DE–01099 Dresden	Haupt	CSP – 25 Aug 2003<br>Amendment no. 1 -<br>4 Febr 2004<br>Amendment no. 2 & 3<br>– 3 May 2005

Centre no.	Name and address of IEC/IRB	Chairman of IEC/IRB	Date of Approval
708	Ärztekammer des Saarlandes<br>Faktoreistr. 4<br>DE–66111 Saarbrücken	Schieffer	CSP – 15 July 2003<br>Amendment no. 1 -<br>23 Febr 2004<br>Amendment no. 2 & 3<br>– 14 April 2005
709	Ärztekammer Berlin<br>Friedrichstr. 16<br>DE–10969 Berlin	C. Jasper<br>NA/Signed by C Biondo	CSP – 25 Aug 2003<br>Amendment no. 1 –<br>10 Febr 2004<br>Amendment no. 2 & 3<br>– 29 april 2005
710	Saechisische Landesaerztekammer, Ethik-Kommission<br>Schuetzenhoehe 16 DE–01099 Dresden	Haupt	CSP – 22 Aug 2003<br>Amendment no. 1 –<br>4 Febr 2004<br>Amendment no. 2 & 3<br>– 3 May 2005
711	Bayerische Landesärztekammer,<br>Ethik-Kommission<br>Muehlbaurstr. 16<br>DE–81677 München	J. Hasford<br>H.Koch<br><br>Beate Henrikus	CSP – 6 Aug 2003<br>Amendment no. 1 -<br>27 Febr 2004<br>Amendment no. 2 & 3<br>– 4 May 2005
713	Ethik-Kommission an der Medizinischen Fakultät der Universität Leipig<br>Härtelstr. 16-18<br>DE–04107 Leipzig	R. Preiss	CSP – 9 Oct 2003<br>Amendment no. 1 -<br>25 Febr 2004<br>Amendment no. 2 & 3<br>– 1 July 2005
715	Zentrum für klinische Studien, Geschäftstelle der Ethik-Kommission der Med. Fakultät der Universität Regensburg<br>Franz Josef Strauss Allee 11<br>DE–93053 Regensburg	R. Andresen	CSP – 20 Oct 2003<br>Amendment no. 1 -<br>27 Jan 2004<br>Amendment no. 2 & 3<br>– 29 April 2005
716	Bayerische Landesärztekammer<br>Ethik-Kommission<br>Muehlbaurstr. 16<br>DE–81677 München	J. Hasford<br>H.Koch<br><br>Beate Henrikus	CSP – 6 Aug 2003<br>Amendment no. 1 -<br>7 Jan 2004<br>Amendment no. 2 & 3<br>– 4 May 2005
717	Ärztekammer Niedersachsen<br>Berliner Allee 20<br>DE–30175 Hannover	K. Held	CSP - 26 aug 2003<br>Amendment no. 1 –<br>2 April 2004<br>Amendment no. 2 & 3<br>– 9 May 2005

Centre no.	Name and address of IEC/IRB	Chairman of IEC/IRB	Date of Approval
719	Ethikkommission Charité<br>Campus Virchow-Klinikum<br>Augustenburger Platz 1<br>DE-13353 Berlin	H Eichstädt	CSP – 18 Febr 2004<br>Amendment no. 1 -<br>17 March 2004
	Charité, Ethikkommission,<br>Ethikausschuss am Campus<br>Virchow-Klinikum,<br>Schumannstr. 20/21 DE–<br>10098 Berlin	E. Garbe	Amendment no. 2 & 3<br>– 20 July 2005
720	Ärztekammer Niedersachsen<br>Berliner Allee 20<br>DE–30175 Hannover	K. Held	CSP - 26 aug 2003<br>Amendment no 1 –<br>2 April 2004<br>Amendment no. 2 & 3<br>– 9 May 2005
723	Ärztekammer Sachsen-Anhalt,<br>Ethik-Kommission<br>Bezirksgeschäftstelle Halle, Am<br>Kirchtor 9<br>DE–06108 Halle	E. Fukala	CSP – 4 Nov 2003<br>Amendment no. 1 -<br>11 March 2004<br>Amendment no. 2 & 3<br>– 6 June 2005
725	Ärztekammer Schleswig-<br>Holstein, Ethik-Kommission<br>Bismarckallee 8-12 DE–<br>23795 Bad Segeberg	H. Hütteroth	CSP – 18 Aug 2003<br>Amendment no. 1 -<br>26 Febr 2004<br>Amendment no. 2 & 3<br>– 17 May 2005
726	Ärztekammer Sachsen-Anhalt,<br>Ethik-Kommission<br>Bezirksgeschäftstelle Halle<br>Am Kirchtor 9<br>DE–06108 Halle	E. Fukala	CSP – 4 Nov 2003<br>Amendment no. 1 –<br>11 March 2004<br>Amendment no. 2 & 3<br>– 6 June 2005
727	Ethik-Kommission bei der<br>Landesärztekammer Hessen<br>Im Vogelgesang 3<br>DE–60488 Frankfurt am Main	A. Helberg-Lubinski	CSP – 13 Aug 2003<br>Amendment no. 1 –<br>16 March 2003<br>Amendment no. 2 & 3<br>– 18 May 2005
729	Ethik-Kommission bei der<br>Landesärztekammer Hessen<br>Im Vogelgesang 3<br>DE–60488 Frankfurt am Main	A. Helberg-Lubinski	CSP – 13 Aug 2003<br>Amendment no. 1 –<br>16 March 2004<br>Amendment no. 2 & 3<br>– 18 May 2005

Centre no.	Name and address of IEC/IRB	Chairman of IEC/IRB	Date of Approval
731	Ärztekammer Schleswig-Holstein, Ethik-Kommission Bismarckallee 8-12 DE–23795 Bad Segeberg	H. Hütteroth	CSP – 18 Aug 2003<br>Amendment no. 1 – 26 Febr 2004<br>Amendment no. 2 & 3 – 17 May 2005
733	Ärztekammer Berlin,<br>Friedrichstr. 16<br>DE–10969 Berlin	C. Jasper<br>NA/Signed by C Biondo	CSP – 25 Aug 2003<br>Amendment no. 1 – 10 Febr 2004<br>Amendment no. 2 & 3 – 29 April 2005
734	EC Georg-August-Universität Göttingen<br>Robert Koch Str. 40<br>DE–37075 Göttingen	Eckart Rüther	CSP – 25 Aug 2003<br>Amendment no. 1 – 28 Jan 2004<br>Amendment no. 2 & 3 – 2 May 2005
737	Ärztekammer Hamburg<br>Heinrich-Hertz Str. 125<br>DE–22083 Hamburg	Weber	CSP – 4 Aug 2003<br>Amendment no. 1 – 26 Jan 2004<br>Amendment no. 2 & 3 – 3 May 2005
743	Universität zu Lübeck<br>Medizinische Fakultät, Ethik-Kommissin<br>Ratzeburger Allee 160<br>DE–23538 Lübeck	Heiner Raspe	CSP – 3 Febr 2004<br>Amendment no. 1 – 24 Febr 2004<br>Amendment no. 2 & 3 – 1 July 2005
744	EC der Med. Fakultät der Ludwig Maximilians Universität München<br>Marchioninistr. 15<br>DE–81377 München	G. Paumgartner	CSP – 1 March 2004<br>Amendment no. 1 – 1 April 2004<br>Amendment no. 2 & 3 – 2 May 2005
746	Ethik-Kommission der Ärztekammer Mecklenburg-Vorpommern bei der Ernst-Moritz-Arndt Universität Greifswald, Medizinische Fakultät, Institut für Pharmakologie<br>Friedrich Löffler Str. 23d<br>DE–17487 Greifswald	W. Siegmund	CSP – 28 Jan 2004<br>Amendment no. 1 – 28 Jan 2004<br>Amendment no. 2 & 3 – 1 June 2005
747	Saechisische Landesaerztekammer, Ethik-Komission, Schuetzenhoehe 16<br>DE–01099 Dresden	Haupt	CSP – 25 Aug 2003<br>Amendment no. 1 – 6 Febr 2004<br>Amendment no. 2 & 3 – 3 May 2005

Centre no.	Name and address of IEC/IRB	Chairman of IEC/IRB	Date of Approval
751	Saechisische Landesaerztekammer, Ethik-Komission, Schuetzenhoehe 16 DE–01099 Dresden	Haupt	CSP – 25 Aug 2003 Amendment no. 1 – 4 Febr 2004 Amendment no. 2 & 3 – 3 May 2005
754	Ärztekammer des Saarlande, Faktoreistr. 4 DE–66111 Saarbrücken	Schieffer	CSP – 15 July 2003 Amendment no. 1 – 7 Jan 2004 Amendment no. 2 & 3 – 14 April 2005
755	Saechisische Landesaerztekammer, Ethik-Komission, Schuetzenhoehe 16 D – 01099 Dresden	Haupt	CSP – 25 Aug 2003 Amendment no. 1 – 4 Febr 2004 Amendment no. 2 & 3 – 3 May 2005
Great Britain All centres	Northern & Yorkshire MREC, John Snow House, Durham University Science Park, Durham DH1 3YG	Jane Lothian<br>Please see Note to File in SMF 3.1.	Amendment no. 1 – 16 Jan 2004 Amendmend 2 & 3 – 12 April 2005
Great Britain 2009	North and Mid Hampshire Local Research Ethics Committees, Harness House, Aldermaston Rd, Basingstoke, Hampshire	Peter Bingham	CSP – 21 Aug 2003
2010-2011	Croydon Local Research EC, C/O Mayday Healthcare NHS Trust, Room 328, Nightingale House, 530 London Rd, Thornton Heath Surrey CR7 7YE	John Chang	CSP – 7 Aug 2003
2012	Mid & South Buckinghamshire Local Research EC, Wycombe hospital, c/o Clinical Effectiveness Dept, Chiltern Medical Educatin Centre, Queen Alexandra Rd, High Wycombe, Buckinghamshire HP11 2TT	Susan Kelly	CSP – 14 Nov 2003
2013	St Thomas' Hospital Research EC, Medical Committee Office, Block 5, South Wing, Lambeth Place Rd, London SE1 7EH	A. Hopper	CSP – 23 Sept 2003

Centre no.	Name and address of IEC/IRB	Chairman of IEC/IRB	Date of Approval
2014	Brighton and Mid Sussex Local Research EC, Brighton and Hove City PCT, 6th Floor, Vantage Point, New England Rd. Brighton, BN1 4GW	Paul Seddon	CSP – 15 Dec 2003
2015	Sandwell & West Birmingham Hospitals NHS Trust, Local Research EC, City Hospital, Dudley Rd Birmingham, B18 7QH, UK	Speculand	CSP – 18 Aug 2003
2017	Greenwich research EC, Healthcare Library, Education Block, Queen Elisabeth Hospital, Stadium Rd, Woolwich, London SE18 4QH	Karen Leball	CSP – 19 aug 2003
2018	South East Wales Local Research EC, Business Services Center, Churchill House, 17 Churchill Way Cardiff CF10 2 TW	D. E. B. Powell	CSP – 4 Sept 2003
2019	Sunderland Local Research EC, Sunderland Teaching Primary Care Trust, Durham Rd, Sunderland, SR3 4AF	J. E. O'Connell	CSP – 22 Dec 2003
2022	Fife Local Research EC, Springfield House, Cupar, Fife KY15 5UP	Esther Roberton	CSP – 7 aug 2003
2023	Brexley Research EC, c/o Healthcare Library, Education Block, Queen Elisabeth Hospital, Stadium Rd, Woolwich London SE18 4QH	Karim Hussain	CSP – 24 Sept 2003
2024	Reasearch EC, Queen's University, Room 1009, Education Centre, Royal Group of Hospitals, Belfast BT12 6BA, Northern Ireland	T. J. Mc Murray	CSP – 19 Aug 2003
2026	National Public Health Services for Wales, Temple of Peace & Health, Cathays Park Cardiff CF10 3NW	Jeremy Winston	CSP – 24 Nov 2003

Centre no.	Name and address of IEC/IRB	Chairman of IEC/IRB	Date of Approval
2027	North East London Health Authority, The Clock House<br>East Street<br>Barking IG11 8EY	Vijay Vasu	CSP – 6 Aug 2003
2029	North East London Strategic Health Authority, Becketts House, 2/14 Ilford Hill<br>Ilford IG1 2QX	N. Akhtar	CSP – 14 Aug 2003
2030	Craigavon Area Hospital Group Trust<br>Trust Headquarters<br>College of Nursing<br>Craigavon Area Hospital<br>68 Lurgan Road<br>Portadown<br>BT63 5QQ	C. Humphrey	CSP – 7 Oct 2003
2031	Dorset Research EC, Poole Hospital NHS Trust, D Block, Room 20, Longfleet Rd,Poole, Dorset BH15 2BJ	Stephanie Wheeler	CSP - 11 Sept 2003
2032	Lanarkshire NHS Board, 14 Beckford Street<br>Hamilton, ML3 0TA	Please see Note to File in SMF 3.1.	CSP - 24 July 2003
2033	East Sussex Local Research EC, Brighton and Hove City PCT, 6th Floor, Vantage Point, New England Rd.<br>Brighton, BN1 4GW	Johan Rademaker	CSP – 3 Dec 2003
2034	West Ethics Committee, Western Infirmary, Dumbarton Rd, Glasgow G11 6NT	Please see Note to File in SMF 3.1.	CSP – 16 Dec 2003
2035	York Research EC, Research Ethics Administration Office, Bootham Park Hospital,<br>York YO30 7BY	Martin Reeder	CSP – 08 aug 2003
2037	South Glasgow University Hospitals NHS Trust, Southern General Hospital, 1345 Govan Rd, Glasgow G51 4TF	D. Keddie	CSP – 27 Jan 2004

Centre no.	Name and address of IEC/IRB	Chairman of IEC/IRB	Date of Approval
2038	County Durham and Darlington, Local Research EC, John Snow House, Durham University Science Park, Durham DH1 3YG	S. Chandler	CSP – 11 Sep 2003
2039	North Sheffield Ethics Office, Northern General Hospital, 1st Floor Vickers Corridor, Herrie Rd, Sheffield, S5 7AU	David Stone	CSP – 20 Aug 2003
2040	Lanarkshire NHS Board, 14 Beckford Street, Hamilton, ML3 0TA	D. Gordon	CSP – 13 Aug 2003
2041. 2043	Liverpool Local Research EC, Hamilton House, 24 Pall Mall, Liverpool L3 6AL	T S Purewal	CSP – 5 Dec 2003
2042	Brompton Harefield & NHLI EC, Royal Brompton Hospital, Sydney Street, London SW3 6NP	D. G. Gibson	CSP – 7 Oct 2003
2045	Leicestershire, Northamptonshire and Rutland Strategic Health Auhority, Lakeside House, 4 Smith Way, Grove Park, Enderby, Leicester LE19 1SS	Peter Rabey	CSP – 5 Sep 2003
2047	Ayrshire & Arran Research EC, Boswell House, 1 Arthur Street, Ayr KA7 1QJ	George L Irving	CSP – 17 Sept 2003
2048	Reasearch EC, Queen’s University, Room 1009, Education Centre, Royal Group of Hospitals, Belfast BT12 6BA, Northern Ireland	T. J. Mc Murray	CSP – 20 Aug 2003
2052	Local Research Ethics Committee Ross House, Hawkhead Road Paisley, PA2 7BN	John G Mullin	CSP – 3 Sept 2003
2054	Local Research EC, Blackpool Victoria Hospital, Trust Headquarters, Whinney Heys Rd, Blackpool, Lancashire FY3 8NR	R. S. Gulati	CSP – 26 Sept 2003

Centre no.	Name and address of IEC/IRB	Chairman of IEC/IRB	Date of Approval
2056	Northern & Yorkshire MREC, John Snow House, Durham University Science Park, Durham DH1 3YG	Please see Note to File in SMF 3.1.	CSP – 24 July 2003
2057	Northern & Yorkshire MREC, John Snow House, Durham University Science Park, Durham DH1 3YG	Please see Note to File in SMF 3.1.	CSP – 24 July 2003
Hungary<br>All centres	Medical Research Council Ethics Committee for Clinical Pharmacology, Arany J. u. 6-8, HU–1051 Budapest	Guayla Papp	CSP – 11 Dec 2003 Amendment no. 1 – 11 Dec 2003 Amendment no. 2 & 3 6 April 2005
Ireland<br>All centres	Irish Medicines Board, Earlsfort Centre, Earlsfort Terrace, Dublin 2, Ireland	Michael Barry	CSP – 15 Jul 2003 Amendment no. 1 – 20 May 2004 Amendment no. 2 & 3 – 21 Sept 2005
The Netherlands<br>All centres	Medical Ethical reviewing committee Noord-Holland, Wilheminalaan 12 NL–1815 JD Alkmaar	P. E. Treffers	CSP – 12 May 2003 Amendment no. 1 – 27 Jan 2004 Amendment no. 2 & 3 – 13 April 2005
The Netherlands<br>1001	Medical Ethical reviewing committee Noord-Holland, Wilheminalaan 12, NL–1815 JD Alkmaar	P. E. Treffers	CSP – 12 May 2003 Amendment no. 1 – 27 Jan 2004 Amendment no. 2 & 3 – 13 May 2005
1002	METC West Brabant, Molengracht 21 NL–4818 CK Breda	A. Stuurman	CSP – 26 Aug 2003 Amendment no. 1 – 27 Jan 2004 Amendment no. 2 & 3 – 13 May 2005
1003	METC MST Haaksbergerstraat 55 NL–7513 ER Enschede	Mr. Bijker	CSP – 2 July 2003 Amendment no. 1 – 27 Jan 2004 Amendment no. 2 & 3 – 5 July 2005
1004	Medisch Ethische Toetsingscommissie Tilburg, Hilvarenbeeksweg 60 NL-5022 GC Tilburg	J. Verheijen-Langenberg	CSP – 17 June 2003 Amendment no. 1 – 27 Jan 2004 Amendment no. 2 & 3 – 13 May 2005

Centre no.	Name and address of IEC/IRB	Chairman of IEC/IRB	Date of Approval
1005	Medisch Ethische Toetsingscommissie, Hanzeplein 1, Postbus 30 001, NL–9700 RB Groningen	J. Davids	CSP – 13 Oct 2003 Amendment no. 1 – 27 Jan 2004 Amendment no. 2 & 3 – 26 May 2005
1006	CMEZ, Oosterscheldeziekenhuis, ‘s-Gravenpolderseweg 114, Postbus 106 NL–4460 BB Goes	H. C. R. Brandenburg	CSP – 22 Aug 2003, Amendment no. 1 – 27 Jan 2004 Amendment no. 2 & 3 – 18 May 2005
1007	Committee Experimental Research on Humans, Tegelseweg 210 NL–5912 BL Venlo	J. A. J. Boermans	CSP – 21 July 2003 Amendment no. 1 – 27 Jan 2004 Amendment no. 2 & 3 – 13 May 2005
1008	Committee TWO, Postbus 1502 NL–3800 BM Amersfoort	Mr. Steeghs	CSP – 4 Sept 2003 Amendment no. 1 – 27 Jan 2004 Amendment no. 2 & 3 – 13 May 2005
1009	METC Reinier de Graafweg 3-11 NL–2625 AD Delft	Mr. Peeters	CSP – 17 Aug 2003 Amendment no. 1 – 27 Jan 2004 Amendment no. 2 & 3 – 11 May 2005
1010	MEC Houtlaan 55 NL–2300 RD Leiden	Mr. Lamping	CSP – 28 Aug 2003 Amendment no. 1 – 27 Jan 2004 Amendment no. 2 & 3 – 17 May 2005
1011	‘t Lange Land Ziekenhuis, Raad van Bestruur, Toneellaan 1, NL–2725 NA Zoetermeer	H. Stokvis	CSP – 24 July 2003 Amendment no. 1 – 27 Jan 2004 Amendment no. 2 - 6 Oct 2005 Amendment no. 3 - 27 May 2005
1012	WMO Kleiweg 500 NL–3004 BA Rotterdadm	C. J. C. Geerlings	CSP – 20 June 2003 Amendment no. 1 – 27 Jan 2004 Amendment no. 2 & 3 – 17 May 2005

Centre no.	Name and address of IEC/IRB	Chairman of IEC/IRB	Date of Approval
1013	METC GZG<br>Tolbrukgstraat 11<br>NL–5211 RW ‘s-Hertogenbosch	M. C. G. Daniles	CSP – 11 Sep 2003<br>Amendment no. 1 – 27 Jan 2004<br>Amendment no. 2 & 3 – 15 May 2005
1014	Clinical Trial Commissie,<br>Ziekenhuis Gooi-Noord,<br>Postbus 900<br>NL–1250 CA Laren	M. J. M. Amsing-Lamers	CSP – 12 Sept 2003<br>Amendment no. 1 – 27 Jan 2004<br>Amendment no. 2 & 3 – 13 May 2005
1015	Medical Ethics Committee,<br>Postbus 98,<br>NL–5700 AB Helmond	A. L. M. Kerremans	CSP – 13 Nov 2003<br>Amendment no. 1 – 18 Febr 2004<br>Amendment no. 2 & 3 – 14 June 2005
1016	Trialbegeleidingscommissie,<br>Postbus 920 NL–<br>6040 Roermond	P. F. Timmermans	CSP – 18 June 2003<br>Amendment no. 1 – 12 Febr 2004<br>Amendment no. 2 & 3 – 23 May 2005
1017	Medisch Etische Commissie,<br>Vlietland Ziekenhuis,<br>Holysingel 3,<br>NL–3136 LA Vlaardingen	H. S. Lau	CSP – 1 July 2003<br>Amendment no. 1 – 27 Jan 2004<br>Amendment no. 2 & 3 – 17 May 2005
1018	Toetsingscommissie<br>Patientgebonden Onderzoek,<br>Banneweg 57<br>NL–4204 AA Gorinchem	T. M. H. Meisters	CSP – 1 Oct 2003<br>Amendment no. 1 – 27 Jan 2004<br>Amendment no. 2 & 3 – 12 May 2005
1019	Medisch Etische<br>Toetsingscommissie,<br>Händellaan 2<br>NL–2100 AJ Heemstede	A. M. M. Clous	CSP – 11 June 2003<br>Amendment no. 1 – 27 Jan 2004<br>Amendment no. 2 & 3 – 19 May 2005
1020	Protocollen<br>Beoordelingscommissie (PBC),<br>Onderdeel van de Medisch<br>Etische Commissie,<br>Boerhaavelan 25,<br>NL–4708 AE Roosendaal	F. van Baars	CSP – 24 June2003<br>Amendment no. 1 – 27 Jan 2004<br>Amendment no. 2 & 3 – 12 May 2005

Centre no.	Name and address of IEC/IRB	Chairman of IEC/IRB	Date of Approval
1021	Local Reviewing committee, Albert Schweitzer hospital, Location Amstelwijck Van der Steenhoven plein 1 NL–3300 AK Dordrecht	C. Pronk	CSP – 2 Sept 2003 Amendment no. 1 – 27 Jan 2004 Amendment no. 2 & 3 – 25 May 2005
1022	Zuiderzeeziekenhuis, Ziekenhuisweg 100 NL–8233 AA Lelystad	J. W. Brinkman	CSP – 19 Nov 2003 Amendment no. 1 – 27 Jan 2004 Amendment no. 2 & 3 – 27 May 2005
1023	Medisch-Etische Toetsingscommissie, Postbus 95500 NL–1090 HM Amsterdam	K. Bloemendaal	CSP – 16 July 2003 Amendment no. 1 – 27 Jan 2004 Amendment no. 2 & 3 – 21 June 2005
1024	Raad am Bestuur, AZM, P. Debyelaan 25 NL–6202 AZ Maastricht	G. J. H. C. M. Peeters	CSP – 1 Dec 2003 Amendment no. 1 – 27 Jan 2004 Amendment no. 2 & 3 – 17 May 2005
1025	Het van Weel-Bethesdda Ziekenhuis Postbus 153 NL–3240 AD Middelharnis	P. C. van der Velden	CSP – 26 Aug 2003 Amendment no. 1 – 27 Jan 2004 Amendment no. 2 & 3 – 12 May 2005
1026	Atrium Medisch Centrum, Henri Dunantstraat 5 NL–6401 CX Heerlen	H. Houben	CSP – 14 Oct 2003 Amendment no. 1 – 27 Jan 2004 Amendment no. 2 & 3 – 11 May 2005
1029	Deventer Ziekenhuis, METC, Postbus 5001 NL–7400 GC Deventer	P. C. Teunissen	CSP – 5 June 2003 Amendment no. 1 – 12 Febr 2004 Amendment no. 2 & 3 – 3 June 2005
1031	Medisch Ethische Toetsingscommissie Noord-Holland, Postbus 501 NL–1800 AM Alkmaar	P. E. Treffers	CSP – 21 July 2003 Amendment no. 1 – 27 Jan 2004 Amendment no. 2 & 3 – 17 May 2005

Centre no.	Name and address of IEC/IRB	Chairman of IEC/IRB	Date of Approval
1032	Medische Etische Toetsingscommissie, Louwesweg 6, Postbus 90440, NL–1006 BK Amsterdam	C. T. J. de Koning	CSP – 2 Sept 2003<br>Amendment no. 1 – 27 Jan 2004<br>Amendment no. 2 & 3 – 27 May 2005
1033	Commissie Toetsings Medische Experimenten, P/a cluster kwaliteitszorg<br>Boerhaavelaan 22<br>NL–2035 RC Haarlem	C. J. M. Adema-de Vries	CSP – 7 July 2003<br>Amendment no. 1 – 27 Jan 2004<br>Amendment no. 2 & 3 – 17 May 2005
1034	Sint Lucas Andreas Ziekenhuis, Medische Ethische toetsingscommissie<br>Postbus 9243,<br>NL–1006 AE Amsterdam	Mieke Commandeur	CSP – 16 July 2003<br>Amendment no. 1 – 27 Jan 2004<br>Amendment no. 2 & 3 – 18 May 2005
1035	Raad van Bestuur, JBZ, Locatie GZG, Nieuwstraat 34<br>NL–5211 NL Den Bosch	F. J. M. Croonen	CSP – 15 Oct 2003<br>Amendment no. 1 – 27 Jan 2004<br>Amendment no. 2 & 3 – 13 May 2005
1036	Medical Ethics Committee, Tergooi Ziekenhuis, Postbus 10016<br>NL–1201 DA Hilversum	F. van Bemmel	CSP – 15 Sept 2003<br>Amendment no. 1 – 27 Jan 2004<br>Amendment no. 2 & 3 – 12 May 2005
1037	Medisch Ethische Toetsingscommissie Twenteborg, Postbus 7600<br>NL–7600 SZ Almelo	H. G. J. Oldenhof	CSP – 07 aug 2003<br>Amendment no. 1 – 27 Jan 2004<br>Amendment no. 2 – 11 May 2205<br>Amendment no. 3 – 20 May 2005
1038	Local Medical Ethical Reviewing Committee<br>Weg door Jonkerbos 100<br>NL–6532 SZ Nijmegen	M. Prick	CSP – 28 Aug 2003<br>Amendment no. 1 – 27 Jan 2004<br>Amendment no. 2 & 3 – 13 May 2005
1039	Raad van Bestuur, Usselland Ziekenhuis,<br>Postbus 690,<br>NL–2900 AR Capelle a/d Ijssel	W. F. Mosselmann	CSP – 12 Aug 2003<br>Amendment no. 1 – 27 Jan 2004<br>Amendment no. 2 & 3 – 17May 2005

Centre no.	Name and address of IEC/IRB	Chairman of IEC/IRB	Date of Approval
1040	Medical Ethics Committee,<br>Postbus 9555,<br>NL–6800 TA Arnhem	A. van Sorge	CSP – 25 Sep 2003<br>Amendment no. 1 –<br>27 Jan 2004<br>Amendment no. 2 & 3<br>– 12 May 2005
1041	Raad van Bestuur, Ziekenhuis<br>Leyenburg<br>Leyweg 275<br>NL–2545 CH Den Haag	R. Treffers	CSP – 21 Aug 2003<br>Amendment no. 1 –<br>27 Jan 2004<br>Amendment no. 2 & 3<br>– 17 May 2005
1042	Medisch Ethische<br>Toetsingscommissie<br>Twenteborg, Postbus 546<br>NL–7550 AM Hengelo	H. G. J. Oldenhof	CSP – 26 Sep 2003<br>Amendment no. 1 –<br>27 Jan 2004<br>Amendment no. 2 & 3<br>– 17 May 2005
1043	Medical Ethics Committee,<br>Postbus 10<br>NL–5340 BE Oss	W. de Boer	CSP – 28 Aug 2003<br>Amendment no. 1 –<br>27 Jan 2004<br>Amendment no. 2 & 3<br>– 27 May 2005
1044	Medisch Ethische Toetsings<br>commissie,<br>Postbus 10400 NL–<br>8000 GK Zwolle	J. C. C. van Niel	CSP – 4 July 2003<br>Amendment no. 1 –<br>27 Jan 2004<br>Amendment no. 2 & 3<br>– 11 May 2005
1045	Medisch Ethische Commissie,<br>Huisduinerweg 3,<br>NL–1782 GZ Den Helder	P. W. H. Houben	CSP – 18 Sep 2033<br>Amendment no. 1 –<br>27 Jan 2004<br>Amendment no. 2 & 3<br>– 3 June 2005
1046	Medisch Ethische<br>Toetsingscommissie, Medisch<br>Centrum Alkmaar, Postbus 501,<br>NL–1800 AM Alkmaar	P. E. Treffers	CSP – 21 July 2003<br>Amendment no. 1 –<br>27 Jan 2004<br>Amendment no. 2 –<br>11 May 2005<br>Amendment no. 3 –<br>1 Aug 2005
1047	Medical Ethical Committee of<br>Hospital Rivierenland Tiel,<br>Pres. Kennedylaan 1, NL–<br>4002 WP Tiel	A. B. Wymenga	CSP – 7 Oct 2003<br>Amendment no. 1 –<br>27 Jan 2004<br>Amendment no. 2 –<br>11 May 2005<br>Amendment no. 3 –<br>23 May 2005

Centre no.	Name and address of IEC/IRB	Chairman of IEC/IRB	Date of Approval
1048	Regionale Toetsingscommissie Patientgebonden Onderzoek, Henri Dunantweg 2 NL–8901 Leeuwarden	Y. E. Van Dijk	CSP – 9 Sep 2003 Amendment no. 1 – 27 Jan 2004 Amendment no. 2 & 3 – 13 May 2005
1049	Medical Ethics Committee, Postbus 80250 NL–3582 KE Utrecht	W. Joosten	CSP – 24 Feb 2004 Amendment no. 1 – 27 Jan 2004 Amendment no. 2 & 3 – 17 May 2005
1050	Raad van Bestuur, Gelre Ziekenhuizen, Sprengenweg 7, NL–7314 ET Apeldoorn	G. J. Heuver	CSP – 08 Sep 2003 Amendment no. 1 – 27 Jan 2004 Amendment no. 2 & 3 – 15 June 2005
1051	Local Reviewing Committee, Hospital Gelderse Vallei, Willy Brandtlaan 10 NL–6710 HN Ede	L. L. Schoots	CSP – 23 Sep 2003 Amendment no. 1 – 27 Jan 2004 Amendment no. 2 & 3 – 11 May 2005
1052	Medical Intstiutional Evaluation Committee, Postbus 20 000 NL–8600 Sneek	W. B. Evers	CSP – 19 April 2004 Amendment no. 1 – 27 Jan 2004 Amendment no. 2 & 3 – 24 May 2005
1053	Local Reviewing Cmmittee, De Run 4600, NL–5504 DB Veldhoven	L. J. J. Derijks	CSP – 10 Aug 2004 Amendment no. 1 – 27 Jan 2004 Amendment no. 2 & 3 – 20 June 2005
Norway All centres	Regional Ethics Committee, Health Region East, Box 1130, Blindern, NO–0318 Oslo	Knut Engedal	CSP – 16 May 2003 Amendment no. 1 – 23 Jan 2004 Amendment no. 2 & 3 – 22 Feb 2005
Poland 1201	Terenova Komisja Bioetyczna Instytucie Kardiologii im. Prymasa Tysiaclecia Stefana, Kardynala Wyszýnskiego, Ul. Alpejska 42 PL–04-628 Warszawa	Tomasz Pasierski	CSP – 2 June 2003 Amendment no. 1 – 26 Jan 2004 Amendment no. 2 & 3 – 7 March 2005

Centre no.	Name and address of IEC/IRB	Chairman of IEC/IRB	Date of Approval
1203	Komisja Bioetyczna przy Akademii Medycznej Ul. Zwirki i Wigury 61, PL–02-097 Warszawa	Aleksander Dubrzýnski	CSP – 10 June 2003 Amendment no. 1 – 16 Dec 2003 Amendment no. 2 & 3 – 22 Feb 2005
1205	Komisja Bioetyczna Okregowej Izby Lekarskiej w Warszawie, Ul. Grojecka 65A, PL–02-094 Warszawa	Marek Czarkowski	CSP – 25 Sep 2003, Amendment no. 1 – 19 Dec 2003 Amendment no. 2 & 3 – 15 Sep 2005
1206	Komisja Bioetyczna Slaskiej Akademii Medycznej, Ul. Warszawska 14, PL–40-006 Katowice	Stefan Kossmann	CSP – 30 Sep 2003 Amendment no. 1 – 3 Feb 2004 Amendment no. 2 & 3 – 8 March 2005
1207	Komisja Bioetyczna Slaskiej Akademii Medycznej, Ul. Warszawska 14, PL–40-006 Katowice	Stefan Kossmann	CSP – 4 Nov 2003 – Amendment no. 1 – 17 Feb 2004 Amendment no. 2 & 3 – 10 May 2005
1208	Komisja Bioetyczna Slaskiej Izby Lekarskiej, Ul. Grazynskiego 49a, PL–40-006 Katowice	Krystyna Sosada	CSP – 29 Sep 2003 Amendment no. 1 - 26 Jan 2004 Amendment no. 2 – Rejected Amendment no. 3 – 28 Feb 2005
1210	Komisja Bioetyki Uniwersytetu Medycznego w Lodzi, Al. Kosciuszki 4 PL–90-419 Lodz	Przedzislaw Polakowski	CSP – 18 Nov 2003 Amendment no. 1 – 18 Nov 2003 Amendment no. 2 & 3 – 14 June 2005
1211	Komisja Bioetyczna przy Akademii Medycznej we Wroclawiu, Ul. Pasteura 1 PL–50-367 Wroclaw	Franciszek Iwanczak	CSP – 12 Juni 2003 Amendment no. 1 – 8 Jan 2004 Amendment no. 2 & 3 – 3 March 2005
1212	Komisja Bioetyczna przy Akademii Medycznej im. Karola Marcinkowskiegow Poznaniu, Ul. Frdry 10 PL–61-701 Poznari	Zygmunt Przybylski	CSP – 6 June 2003 Amendment no. 1 – 8 Jan 2004 Amendment no. 2 & 3 – 10 March 2005

Centre no.	Name and address of IEC/IRB	Chairman of IEC/IRB	Date of Approval
1213	Komisja Bioetyczna Okregowej Rady Lekarskiej w Bialymstoku, Ul. Swietojanska 7 PL–15-082 Bialystok	Wojciech Pedich	CSP – 2 July 2003 Amendment no. 1 – 10 Dec 2003 Amendment no. 2 & 3 – 2 March 2005
1214	Komisja Bioetyczna Okregowej Izby Lekarskiej w Warszawie, Ul. Grojecka 65a PL–02-094 Warszawa	Marek Czarkowski	CSP – 25 Sep 2003 Amendment no. 1 – 19 dec 2003 Amendment no. 2 & 3 – 15 Sep 2005
1215	Komisja Bioetyczna Okregowej Izby Lekarskiej w Warszawie, Ul. Grojecka 65a PL–02-094 Warszawa	Marek Czarkowski	CSP – 25 Sep 2003 Amendment no. 1 – 19 Dec 2003, Amendment no. 2 & 3 – 15 Sep 2005
1216	Komisja Bioetyczna pryzy Okregowej Izbie Lekarskiej w Lodzi, Ul. Czerwona 3 PL–93-058 Lodz	Andrzej Klimek	CSP – 23 July 2003 Amendment no. 1 – 10 Dec 2003 Amendment no. 2 & 3 – 16 March 2005
1217	Komisja Bioetyczna pryzy Okregowej Izbie Lekarskiej w Lodzi, Ul. Czerwona 3 PL–93-058 Lodz	Andrzej Klimek	CSP – 24 Sep 2003 Amendment no. 1 – 10 Dec 2003 Amendment no. 2 & 3 – 16 March 2005
1218	Komisja Bioetyczna pryzy Okregowej Izbie Lekarskiej w Lodzi, Ul. Czerwona 3 PL–93-058 Lodz	Andrzej Klimek	CSP – 24 Sep 2003 Amendment no. 1 – 10 Dec 2003 Amendment no. 2 & 3 – 16 March 2005
1219	Komisja Bioetyczna Bydgoskiej Izby Lekarskiej, Ul. Powstancow Warszawy 11 PL–85-681 Bydgoszcz	Wladyslaw Sinkiewicz	CSP – 14 Oct 2003 Amendment no. 1 – 16 March 2004, Amendment no. 2 & 3 – 12 April 2004
1220	Komisja Bioetyczna Switokrzyskiej Izby Lekarskiej, Ul. Wojska Polskiego 52 PL–25-389 Kielce	Krzysztof Bartosz	CSP – 7 Oct 2003 Amendment no. 1 – 13 Jan 2004 Amendment no. 2 & 3 – 29 March 2005

Centre no.	Name and address of IEC/IRB	Chairman of IEC/IRB	Date of Approval
1221	Komisja Bioetyczna Okregowej Rady Lekarskiej w Lublinie, ul. Chmielna 4<br>PL–20-079 Lublin	Ewa Tuszkiewicz-Misztral	CSP – 2 sept 2003<br>Amendment no. 1 –<br>3 Feb 2004<br>Amendment no. 2 & 3 – 29 March 2005
1224	Komisja Bioetyczna Dolnoslaskiej Izby Lekarskiej we Wroclawiu, ul. Matejki 6<br>PL–50-333 Wroclaw	Wlodzimierz Bednorz	CSP – 25 June 2003<br>Amendment no. 1 –<br>21 Jan 2004<br>Amendment no. 2 & 3 – 13 April 2005
1227	Komisja Bioetyczna pryzy Okregowej Izbie Lekarskiej w Gdansku, Ul. Norwida 4 PL–81-434 Gdynia	Jerzy Umiastowski<br>Henryk Mionskowski	CSP – 23 Oct 2003<br>Amendment no. 1 –<br>25 March 2004<br>Amendment no. 2 & 3 – 3 March 2005
1229	Komisja Bioetyczna pryzy Okregowej Izbie Lekarskiej w Gdansku, Ul. Norwida 4 PL–81-434 Gdynia	Jerzy Umiastowski<br>Henryk Mionskowski	CSP – 23 Oct 2003<br>Amendment no. 1 –<br>25 March 2004<br>Amendment no. 2 & 3 – 3 March 2005
1230	Komisja Bioetyczna przy Wojskowej Izbie Lekarskiej, Ul. Szaserow 128<br>PL–00-909 Warszawa	Dariusz Jurkiewicz	CSP – 19 Nov 2003<br>Amendment no. 1 –<br>3 Dec 2003<br>Amendment no. 2 & 3 – 24 April 2005
1231	Komisja Bioetyczna przy Warminsko-Mazurskiej Izbie Lekarskej w Olsztynie, ul. Zolnierska 16<br>PL–10-561 Olsztyn	Zdzislaw Piesiak	CSP – 20 Nov 2003<br>Amendment no. 1 –<br>24 Feb 2004<br>Amendment no. 2 & 3 – 17 May 2005
1232	Komisja Bioetyczna przy Bydgoskiej Izbie Lekarskiej w Bydgoszczy, Ul. Powstancow Warszawy 11<br>PL–85-681 Bydgoszcz	Emilia Pietkiewicz	CSP – 18 Nov 2003,<br>Amendment no. 1 –<br>16 March 2004<br>Amendment no. 2 & 3 – 12 April 2005
1233	Komisja Bioetyczna Slaskiej Izby Lekarskiej w Katowicach, Ul. Grazynskiego 49a<br>PL-40-126 Katowice	Krystyna Sosada	CSP – 24 Nov 2003<br>Amendment no. 1 –<br>26 Jan 2004,<br>Amendment no. 2 – Rejected. Amendment no. 3 – 28 Feb 2005

Centre no.	Name and address of IEC/IRB	Chairman of IEC/IRB	Date of Approval
Portugal<br>901	Comissão de Ética do Hospital S. Fransisco Xavier, Estrada Forte do Alto do Duque PT–1440-005 Lisboa	Carlos Neves	CSP & Amendment no. 1 – 7 June 2004 Amendment no. 2 & 3 – 27 June 2005
903	Comissão de Ética do Hospital Amadra sintra, S.A Estrada IC19, Mina PT–2700-276 Amadora	Rocha Pires<br><br>Luis Afonso Dutschmann	CSP and Amendment no. 1– 2 March 2004 Amendment no. 2 & 3 - 30 June 2005
905	Comissão de Ética do Hospital de Santa Maria, Av. Prof. Egas Moniz PT–1649-035 Lisboa	Frederico Silveira Machado	CSP & Amendment no. 1 – 25 March 2004 Amendment no. 2 & 3 – 9 Nov 2005
906	Comissão de Ética do Hospital Central do Funchal, Av, Luís Camões, S. Pedro PT–9000 Funchal	Edward Richard Rushworth Maul	CSP & Amendment no. 1 – 24 March 2004 Amendment no. 2 & 3 – 2 March 2005
907	Comissão de Ética do Hospital Nossa Sra Do Rosário, Av. DasForcas Armadas PT–2830 Barreiro	José Pereira	CSP & Amendment no. 1 – 2 March 2004 Amendment no. 2 & 3 - 5 Feb 2005
909	Comissão de Ética do Hospital Reynaldo dos Santos, R. Dr. Luís César Pereira PT–2601-909 Vila Franca de Xira	Sieuve Afonso<br><br>João Franca Gouveia	CSP & Amendment no. 1 – 7 April 2004 Amendment no. 2 & 3 – 10 March 2005
912	Comissão de Ética do Hospital Padre Américo/Vale do Sousa, Penafiel- Guilhufe PT–4560-162 Guilhufe	Braga da Cunha	CSP& Amendment no. 1 – 23 April 2004 Amendment no. 2 & 3 – 18 March 2005
Romania<br>All centres	National Ethics Committee for the Clinical Study of the Drugs, Bucharest, Str.Aviator Sanatescu, no.48 RO-71324 Bucuresti	G Litarczek	CSP – 31 July 2003 Amendment no. 1 – 30 Jan 2004 Amendment no. 2 & 3 – 18 March 2005
Russia<br>1401, 1402, 1403	Clinical Cardiology Ethics Committee, 15A, 3-rd Cherepkovskaya str. RU–121552 Moscow	M.Y Ruda	CSP & Amendment no. 1 – 29 Dec 2003 Amendment no. 2 & 3 – 27 June 2005

Centre no.	Name and address of IEC/IRB	Chairman of IEC/IRB	Date of Approval
1404	Ethics Committee of City Hospital No. 59, 31/33, Dostoevskogo str. RU–127143 Moscow	D.P Semerntsov	CSP & Amendment no. 1 – 22 April 2004 Amendment no. 2 & 3 – 31 May 2005
1405	IEC of Educational Scientific Centre of Medical Centre of President Affairs Management, Timoshenko str. 21 RU–121356 Moscow	V.N Semenov<br>D.A Zateysheichikov	CSP & Amendment no. 1 – 15 Dec 2003 Amendment no. 2 & 3 – 8 June 2005
1406	Ethics Committee of Out-patient Clinic no. 1, 31, Grokholsky str. RU–129010, Moscow	E.S Begotskaya	CSP & Amendment no. 1 – 28 Nov 2003 Amendment no. 2 & 3 – 27 May 2005
1407	IEC of Medical Stomatology University, Delegatskaya str. 20/1, RU–Moscow	A.L Davidov	CSP & Amendment no. 1 – 8 Dec 2003 Amendment no. 2 & 3 – 30 May 2005
1408	Ethics Committee of Russian State Medical University, 1, Ostrovitjanova str. RU–117997 Moscow	G.I Strozhakov	CSP – 24 Nov 2003 Amendment no. 1 – 22 Dec 2003 Amendment no. 2 & 3 – 20 June 2005
1409	IEC of Military Institute of Postgraduated Education of Doctors of Russian Federation, Hospitalnaya square 3, RU–105229 Moscow	L.V Pisarenko	CSP & Amendment no. 1 – 10 Dec 2003 Amendment no. 2 & 3 – 20 May 2005
1410	Ethics Committee of Medical Faculty of Russian People Friendship University, 8/1, Miklukho-Maklaya str. RU–117198 Moscow	Y.V Tarichko	CSP & Amendment no. 1 – 12 March 2004 Amendment no. 2 & 3 – 16 May 2005
1411	Ethics Committtee of State Institution State Research Institute of Pyysico-Chemical Medicine of MoH of Russian Federation, Hospital Square 2, RU–111020 Moscow	N.A Vaulin	CSP & Amendment no. 1 – 13 april 2004 Amendment no. 2 & 3 – 24 June 2005
1412	Ethics Committee of Russian State Medical University, 1, Ostrovitjanova str. RU–117997 Moscow	G.I Strozhakov	CSP & Amendment no. 1 – 17 May 2004 Amendment no. 2 & 3 – 20 June 2005

Centre no.	Name and address of IEC/IRB	Chairman of IEC/IRB	Date of Approval
Slovakia<br>1501	Etická komisia pri Nemocnici s polklinikou<br>Ruzinovská 6,<br>SK–826 06 Bratislava	Magdaléna Fuceková<br>Anna Krechnáková	CSP – 11 June 2003<br>Amendment no. 1 – 28 Jan 2004<br>Amendment no. 2 – 24 May 2005<br>Amendment no.3 – 17 Feb 2005
1502	Etická komisia lekárskej fakulty a fakultnej nemocnice UK,<br>Mickiewiczova 13,<br>SK-813 69 Bratislava	Jan Porubský	CSP – 23 June 2003<br>Amendment no. 1 – 8 Dec 2003<br>Amendment no. 2 – 23 May 2005<br>Amendment no. 3 – 14 March 2005
1503	Etická komisia lekárskej fakulty a fakultnej nemocnice UK,<br>Mickiewiczova 13,<br>SK-813 69 Bratislava	Jan Porubský	CSP – 22 Sep 2003<br>Amendment no. 1 – 19 Jan 2004<br>Amendment no. 2 – 20 June 2005<br>Amendment no. 3 – 14 March 2005
1504	Ethics committee of the SÙSCH, Pod Krásnou hôrkou 1,<br>SK–833 48 Bratislava	Eva Silvanová<br>Etela Janeková	CSP – 2 July 2003<br>Amendment no. 1 – 25 Feb 2004<br>Amendment no. 2 – 15 June 2005<br>Amendment no. 3 – 14 March 2005
1505	Etická komisia pri Nemocnici s poliklinikou,<br>Spitálska 6, PP41C<br>SK–949 01 Nitra	Miroslav Murgas	CSP – 27 June 2003<br>Amendment no. 1 – 8 Jan 2004<br>Amendment no. 2 – 24 June 2005<br>Amendment no. 3 – 15 April 2005
1506	Etická komisia pri Nemocnici s poliklinikou,<br>Spitálska 6, PP41C<br>SK–949 01 Nitra	Miroslav Murgas	CSP – 20 June 2003<br>Amendment no. 1 – 8 Jan 2004<br>Amendment no. 2 – 24 June 2005<br>Amendment no. 3 – 15 April 2005

Centre no.	Name and address of IEC/IRB	Chairman of IEC/IRB	Date of Approval
1507	Etická komisia pri Nemocnici s poliklinikou<br>Slovenská 11<br>SK–94030 Nové Zámky	Ján Bernát	CSP – 10 June 2003<br>Amendment no. 1 – 27 Jan 2004<br>Amendment no. 2 – 2 June 2005<br>Amendment no. 3 – 23 March 2005
1509	Etická komisia pri NsP L. N. Jégeho, Nemocnicna 1, SK–026 14 Dolný Kubín	Agnesa Bjelová	CSP – 16 June 2003<br>Amendment no. 1 – 29 March 2004
	Etická komisia Zilinského samosprávneho kraja<br>Janka Kalinciaka 14,<br>SK–011 09 Zlina	Martin Kapasný	Amendment no. 2 & 3 – 20 Sept 2005
1510	Etická komisia pri NsP J.A. Reimana,<br>Holého 14<br>SK–081 01 Presov	Vladimír Goc	CSP – 10 June 2003<br>Amendment no. 1 – 11 Dec 2003<br>Amendment no. 2 – 9 May 2005<br>Amendment no. 3 – 23 Feb 2005
1511	Etická komisia FNLP,<br>Ratislavova 43<br>SK–041 90 Kosice	Mária Wagnerová<br><br>Rafael Rybár	CSP – 17 June 2003<br>Amendment no. 1 – 4 Dec 2003<br>Amendment no. 2 – 14 June 2005<br>Amendment no. 3 – 14 March 2005
1512	Etická komisia Východoslovenského ústavu srdcových chorôb, Tr. SNP 1,<br>SK–040 66 Kosice	Stanislav Juhás	CSP – 1 July 2003<br>Amendment no. 1 – 17 March 2004<br>Amendment no. 2 – Rejected<br>Amendment no. 3 – 15 April 2005
1513	Etická komisia pri NsP MV SR, ul. Frana Král’a 14<br>SK–812 72 Bratislava	Daniela Harustiaková<br><br>Vladimír Majtán	CSP – 25 June 2003<br>Amendment no. 1 – 6 Feb 2004<br>Amendment no. 2 – 9 June 2005<br>Amendment no. 3 – 15 March 2005

Centre no.	Name and address of IEC/IRB	Chairman of IEC/IRB	Date of Approval
1515	Etická komisia pri NsP, Nám. Republiky 14, SK–984 39 Lucenec	Július Höffer	CSP & Amendment no. 1 – 29 Jan 2004<br>Amendment no. 2 – 26 May 2005
		Ládislav Misanik	Amendment no. 3 – 3 March 2005
1516	Etická komisia Nemocnica s políklinikou Sv. Jakuba, Bardejov, Sv. Jakuba 21, SK-085 76 Bardejov	Martina Suchová	CSP & Amendment no. 1 – 10 Feb 2004<br>Amendment no. 2 – 16 May 2005<br>Amendment no. 3 – 15 Feb 2005
South Africa<br>1601	University of Stellenbosch, Faculty of Health Science, Division of Research Development and Support, PO Box 19063, Tygerberg 7505, ZA	List of members	CSP – 15 Aug 2003<br>Amendment no. 1 – 9 Dec 2003<br>Amendment no. 3 – 10 March 2005
1602	University of the Witwatersrand, Human Research Ethics Committee, Senate House, 1 Jan Smuts Avenue, Braamfontein, Johannesburg 2000, ZA	Maureen Joffe	CSP – 14 Oct 2003<br>Amendment no. 1 – 4 Dec 2003<br>Amendment no. 3 – 12 April 2005
1603, 1604, 1605, 1607	Pharma-Ethics Ltd, 123 Amcor Road, Lyttelton Manor 0157, ZA	L. Venter	CSP – 23 May 2003<br>Amendment no. 1 – 4 Feb 2004<br>Amendment no. 3 – 20 April 2005
1606	University of the Free State, Faculty of Health Sciences, 339 Bloemfontein 9300, ZA	B. B. Hoek	CSP – 27 May 2003<br>Amendment no. 1 - 27 Jan 2004<br>Amendment no. 3 – 15 March 2005
South Africa All centres			Amendment no. 2 – Not applicable
Spain<br>1701	CAEC Consejería de Salud Avda. De la Innovación, s/n Edificio Arena, ES–41020 Sevilla	Isabel Fernández Fernández	CSP & Amendment no. 1 – 12 March 2004<br>Amendment no. 2 – Rejected<br>Amendment no. 3 – 1 March 2005

Centre no.	Name and address of IEC/IRB	Chairman of IEC/IRB	Date of Approval
1703	Comité Ético de Investigación Clínica Vall d'Hebron, Pg. Vall d'Hebron 119-129,<br>ES–08035 Barcelona	José Bruno Montoro	CSP – 7 July 2003<br>Amendment no. 1 –<br>1 March 2004<br>Amendment no 2 & 3 – 1 March 2005
1704	CAEC Consejería de Salud<br>Avda. De la Innovación, s/n<br>Edificio Arena<br>ES–41020 Sevilla	Isabel Fernández Fernández	CSP & Amendment no. 1 – 12 March 2004<br>Amendment no. 2 – Rejected<br>Amendment no. 3 –<br>1 March 2005
1705	CEIC Hospital Clinic I Provincial de Barcelona,<br>Villaroel 170<br>ES–08036 Barcelona	R. Gomis	CSP – 3 June 2003<br>Amendment no. 1 –<br>14 Jan 2004<br>Amendment no. 2 & 3 – 10 Febr 2005
1706	CEIC Regional de Madrid<br>Unidad de Bioetica,<br>Viceonsejeria de Ordenacion,<br>Sanitaria y Salud Publica,<br>c/Aduana, 29 3° Planta<br>ES-28013 Madrid	Francisco Abad Santos	CSP – 11 May 2003<br>Amendment no. 1 –<br>26 Jan 2004<br>Amendment no. 2 & 3<br>10 March 2005
1707	Comité Ético de Investigación Clínica Edificio Administrativo San Lázaro,<br>ES–15703 Santiago de Compostela	Miguel Amor Otero	CSP – 16 June 2003<br>Amendment no. 1 –<br>11 March 2004<br>Amendment no. 2 & 3 – 23 Feb 2005
1708	CEIC Regional de Madrid<br>Unidad de Bioetica,<br>Viceonsejeria de Ordenacion,<br>Sanitaria y Salud Publica,<br>c/Aduana, 29 3° Planta<br>ES-28013 Madrid	Francisco Abad Santos	CSP – 11 May 2003<br>Amendment no. 1 –<br>26 Jan 2004<br>Amendment no. 2 & 3<br>10 March 2005
1709	CEIC Regional de Madrid<br>Unidad de Bioetica,<br>Viceonsejeria de Ordenacion,<br>Sanitaria y Salud Publica,<br>c/Aduana, 29 3° Planta<br>ES-28013 Madrid	Francisco Abad Santos	CSP – 11 May 2003<br>Amendment no. 1 –<br>26 Jan 2004<br>Amendment no. 2 & 3<br>10 March 2005

Centre no.	Name and address of IEC/IRB	Chairman of IEC/IRB	Date of Approval
1716	CAEC Consejería de Salud Avda. De la Innovación, s/n Edificio Arena ES–41020 Sevilla	Isabel Fernández Fernández	CSP & Amendment no. 1 – 12 March 2004 Amendment no. 2 – Rejected Amendment no. 3 – 1 March 2005
1717	CEIC Hospital Universitari Sant Joan d’Alacant, Ctra Nacional 332 Alicante-Valencia s/n San Juan de Alicante, ES–03550 Alicante	José Lizón Giner	CSP – 23 Sep 2003 Amendment no. 1 – 17 Febr 2004 Amendment no. 2 & 3 – 22 Feb 2005
1718	CEIC Hospital Donosita, Plaza Dr. Beguiristain s/n, ES–20014 Donostia – San Sebastian	Montserrat Clerigué	CSP – 1 July 2003 Amendment no. 1 – 14 Jan 2004 Amendment no. 2 & 3 – 24 Feb 2005
1719	CEIC Hospital Mutua de Terrassa, Plaza dr. Robert 5 ES–08221 Terrassa	Ramón Pla Poblador	CSP – 28 May 2005 Amendment no. 1 – 28 Jan 2004 Amendment no. 2 & 3 – 1 April 2005
1721	CAEC Consejería de Salud Avda. De la Innovación, s/n Edificio Arena ES–41020 Sevilla	Isabel Fernández Fernández	CSP & Amendment no. 1 – 12 March 2004 Amendment no. 2 – Rejected Amendment no. 3 – 1 March 2005
1723	CEIC Illes Balears Cecili Metel 18 ES–07003 Palma de Mallorca	Guillem Frontera Juan Olga Delgado Sanchez	CSP – 24 Sep 2003 Amendment no. 1 – 28 Jan 2004 Amendment no. 2 & 3 – 23 Feb 2005
1724	Comité Ético de Investigación Clínica Edificio Administrativo San Lázaro, ES–15703 Santiago de Compostela	Miguel Amor Otero	CSP – 16 June 2003 Amendment no. 1 – 11 March 2004 Amendment no. 2 & 3 – 23 Feb 2005

Centre no.	Name and address of IEC/IRB	Chairman of IEC/IRB	Date of Approval
1725	CEIC Hospital Universitario de Salamanca, Pase de San Vicente 58-182,<br>ES–37007 Salamanca	Concepción Ceballos Alonso	CSP & Amendment no. 1 – 19 jan 2004<br>Amendment no. 2 – 21 Febr 2005<br>Amendment no. 3 – 1 March 2005
1726	Comité Ético de Investigación Clinica, Hospital Unversitario Marqués de Valdecilla, Avda, Marqués de Valdecilla, s/n,<br>ES–39008 Santander	Felipe de la Llama Vásquez	CSP & Amendment no. 1 – 28 April 2004<br>Amendment no. 2 & 3 – 18 Feb 2005
1728	Comité Ético de Investigación Clínica Edificio Administrativo San Lázaro,<br>ES–15703 Santiago de Compostela	Miguel Amor Otero	CSP – 16 June 2003<br>Amendment no. 1 – 11 March 2004<br>Amendment no. 2 & 3 – 23 Feb 2005
Sweden<br>1801-1813, 1815, 1817, 1819, 1820, 1822,	Göteborgs Universitet, Medicinska Fakultetens Forskningskommitté, Box 454, SE–405 30 Göteborg	Calle Bengtsson	CSP – 16 Sep 2003<br>Amendment no. 1 – 9 Dec 2003
1814, 1816	Göteborgs Universitet, Medicinska Fakultetens Forskningskommitté, Box 454, SE–405 30 Göteborg	Calle Bengtsson	CSP – 16 Oct 2003<br>Amendment no. 1 – 9 Dec 2003
1821	Göteborgs Universitet, Medicinska Fakultetens Forskningskommitté, Box 454, SE–405 30 Göteborg	Calle Bengtsson	CSP – 15 Oct 2003<br>Amendment no. 1 – 9 Dec 2003
1825, 1826, 1827	Göteborgs Universitet, Medicinska Fakultetens Forskningskommitté, Box 454, SE–405 30 Göteborg	Calle Bengtsson	CSP – 20 Nov 2003<br>Amendment no. 1 – 9 Dec 2003
All centres	Regionala Etikprövningsnämnden,<br>Box 100<br>SE–405 30 Göteborg	Margareta Möller	Amendment no. 2 – 6 July 2005<br>Amendment no. 3 – 10 Feb 2005

Centre no.	Name and address of IEC/IRB	Chairman of IEC/IRB	Date of Approval
Switzerland<br>1901	Comite d'etique du departement de medicine, Hopitaux universitaries<br>CH–1211 Geneve	R. Rizzoi	CSP – 27 Aug 2003<br>Amendment no. 1 – 14 Jan 2003<br>Amendment no. 2 – Rejected<br>Amendment no. 3 – 1 June 2005
1903	Comitato Etico Cantonale<br>CH–6501 Bellinzona	C. Marone<br><br>M Zanini	CSP – 9 Dec 2003<br>Amendment no. 1 – 28 Jan 2003<br>Amendment no. 2 & 3 – 25 July 2005
1905	Kantonale Ethikkommission<br>CH–8090 Zürich	R. Maurer	CSP & Amendment no. 1 – 19 Dec 2003<br>Amendment no. 2 & 3 – 22 April 2005

12.1.3.2 Samples of written Subject Information and Consent Form

Version of Informed Consent Form	Date of issue
Sample Written Informed Consent	28 October 2002
Genetic Research Addendum to Informed Consent Form	15 October 2004
Adult Study Subject Information and Consent Form	16 December 2004
